# Core outcome sets for trials of interventions to prevent and to treat multimorbidity in adults in low and middle-income countries: the COSMOS study

**DOI:** 10.1136/bmjgh-2024-015120

**Published:** 2024-08-19

**Authors:** Aishwarya Lakshmi Vidyasagaran, Rubab Ayesha, Jan R Boehnke, Jamie Kirkham, Louise Rose, John R Hurst, Juan Jaime Miranda, Rusham Zahra Rana, Rajesh Vedanthan, Mehreen Riaz Faisal, Saima Afaq, Gina Agarwal, Carlos Alberto Aguilar-Salinas, Kingsley Akinroye, Rufus Olusola Akinyemi, Syed Rahmat Ali, Rabeea Aman, Cecilia Anza-Ramirez, Koralagamage Kavindu Appuhamy, Se-Sergio Baldew, Corrado Barbui, Sandro Rogerio Rodrigues Batista, María del Carmen Caamaño, Asiful Haidar Chowdhury, Noemia Teixeira de Siqueira-Filha, Darwin Del Castillo Fernández, Laura Downey, Oscar Flores-Flores, Olga P García, Ana Cristina García-Ulloa, Richard IG Holt, Rumana Huque, Johnblack K Kabukye, Sushama Kanan, Humaira Khalid, Kamrun Nahar Koly, Joseph Senyo Kwashie, Naomi S Levitt, Patricio Lopez-Jaramillo, Sailesh Mohan, Krishna Prasad Muliyala, Qirat Naz, Augustine Nonso Odili, Adewale L Oyeyemi, Niels Victor Pacheco-Barrios, Devarsetty Praveen, Marianna Purgato, Dolores Ronquillo, Kamran Siddiqi, Rakesh Singh, Phuong Bich Tran, Pervaiz Tufail, Eleonora P Uphoff, Josefien van Olmen, Ruth Verhey, Judy M Wright, Jessica Hanae Zafra-Tanaka, Gerardo A Zavala, Yang William Zhao, Najma Siddiqi, on behalf of the COSMOS collaboration, Jibril Abdulmalik

**Affiliations:** 1 Department of Health Sciences, University of York, York, UK; 2 Institute of Psychiatry, Rawalpindi Medical University, Rawalpindi, Pakistan; 3 Foundation University School of Science and Technology, Rawalpindi, Pakistan; 4 School of Health Sciences, University of Dundee, Dundee, UK; 5 Centre for Biostatistics, The University of Manchester, Manchester, UK; 6 Manchester Academic Health Science Centre, Manchester, UK; 7 Faculty of Nursing, Midwifery and Palliative Care, King’s College London, London, UK; 8 UCL Respiratory, University College London, London, UK; 9 CRONICAS Center of Excellence in Chronic Diseases, Universidad Peruana Cayetano Heredia, Lima, Peru; 10 Faculty of Medicine and Health, Sydney School of Public Health, University of Sydney, Sydney, New South Wales, Australia; 11 The Healing Triad Pakistan, Lahore, Pakistan; 12 Section for Global Health, Department of Population Health, NYU Grossman School of Medicine, New York, NY, USA; 13 Institute of Public Health and Social Sciences, Khyber Medical University, Peshawar, Pakistan; 14 Department of Epidemiology and Biostatistics, Imperial College London, London, UK; 15 Department of Family Medicine, Faculty of Health Sciences, McMaster University, Hamilton, Ontario, Canada; 16 Instituto Nacional de Ciencias Médicas y Nutrición Salvador Zubirán, Mexico City, Mexico; 17 Nigerian Heart Foundation, Lagos, Nigeria; 18 Neuroscience and Ageing Research Unit, Institute for Advanced Medical Research and Training, University of Ibadan, Ibadan, Nigeria; 19 Khyber Medical University, Peshawar, Pakistan; 20 Foundation University Islamabad, Islamabad, Pakistan; 21 Physical Therapy Department, Anton de Kom University of Suriname, Paramaribo, Suriname; 22 Department of Neurosciences, Biomedicine and Movement Sciences, Section of Psychiatry, University of Verona, Verona, Italy; 23 Family Medicine and Primary Health Care, Universidade Federal de Goiás, Goiania, Brazil; 24 Facultad de Ciencias Naturales, Universidad Autónoma de Querétaro, Queretaro, Mexico; 25 ARK Foundation, Dhaka, Bangladesh; 26 Department of Global Health, University of Washington, Seattle, Washington, USA; 27 The George Institute for Global Health, University of New South Wales, Sydney, New South Wales, Australia; 28 The George Institute for Global Health, Imperial College London, London, UK; 29 Centro de Investigación del Envejecimiento (CIEN), Facultad de Medicina Humana, Universidad de San Martin de Porres, La Molina, Peru; 30 Facultad de Ciencias de la Salud, Universidad Cientifica del Sur, Lima, Peru; 31 Centro de Atención Integral del Paciente con Diabetes (CAIPaDi), Instituto Nacional de Ciencias Medicas y Nutricion Salvador Zubiran, Mexico City, Mexico; 32 Human Development and Health, Faculty of Medicine, University of Southampton, Southampton, UK; 33 Research and development, ARK Foundation, Dhaka, Bangladesh; 34 Department of Economics, University of Dhaka, Dhaka, Bangladesh; 35 Uganda Cancer Institute, Kampala, Uganda; 36 SPIDER, Department of Computer and Systems Sciences, Stockholm University, Stockholm, Sweden; 37 Pakistan Institute of Living and Learning, Karachi, Pakistan; 38 Health System and Population Studies Division, International Centre for Diarrhoeal Disease Research, Bangladesh (ICDDR,B), Dhaka, Bangladesh; 39 Community and Family Aid Foundation, Accra, Ghana; 40 Division of Endocrinology, Department of Medicine, University of Cape Town, Cape Town, South Africa; 41 Masira Research Institute, Universidad de Santander, Bucaramanga, Colombia; 42 Facultad de Ciencias Medicas Eugenio Espejo, Universidad UTE, Quito, Ecuador; 43 Public Health Foundation of India, New Delhi, India; 44 Centre for Chronic Disease Control (CCDC), New Delhi, India; 45 National Institute of Mental Health and Neurosciences (NIMHANS), Bangalore, Karnataka, India; 46 Institute of Psychiatry, Benazir Bhutto Hospital, Rawalpindi, Pakistan; 47 Circulatory Health Research Laboratory, University of Abuja College of Health Sciences, Abuja, Nigeria; 48 College of Health Solutions, Arizona State University, Tempe, Arizona, USA; 49 Department of Physiotherapy, University of Maiduguri, Maiduguri, Nigeria; 50 The George Institute for Global Health India, Hyderabad, India; 51 Hull York Medical School, Hull, UK; 52 Department of Public Health, KIST Medical College, Kathmandu, Nepal; 53 Department of Family Medicine and Population Health, University of Antwerp, Antwerpen, Belgium; 54 Private Researcher, New York, NY, USA; 55 Centre for Reviews and Dissemination, University of York, York, UK; 56 Family Medicine and Population Health, University of Antwerp, Antwerpen, Belgium; 57 The Friendship Bench, Harare, Zimbabwe; 58 Leeds Institute of Health Sciences, University of Leeds, Leeds, UK; 59 The George Institute for Global Health, Peking University Health Science Centre, Beijing, People's Republic of China

**Keywords:** Prevention strategies, Treatment, Other study design

## Abstract

**ABSTRACT:**

**Introduction:**

The burden of multimorbidity is recognised increasingly in low- and middle-income countries (LMICs), creating a strong emphasis on the need for effective evidence-based interventions. Core outcome sets (COS) appropriate for the study of multimorbidity in LMICs do not presently exist. These are required to standardise reporting and contribute to a consistent and cohesive evidence-base to inform policy and practice. We describe the development of two COS for intervention trials aimed at preventing and treating multimorbidity in adults in LMICs.

**Methods:**

To generate a comprehensive list of relevant prevention and treatment outcomes, we conducted a systematic review and qualitative interviews with people with multimorbidity and their caregivers living in LMICs. We then used a modified two-round Delphi process to identify outcomes most important to four stakeholder groups (people with multimorbidity/caregivers, multimorbidity researchers, healthcare professionals and policymakers) with representation from 33 countries. Consensus meetings were used to reach agreement on the two final COS. Registration: https://www.comet-initiative.org/Studies/Details/1580.

**Results:**

The systematic review and qualitative interviews identified 24 outcomes for prevention and 49 for treatment of multimorbidity. An additional 12 prevention and 6 treatment outcomes were added from Delphi round 1. Delphi round 2 surveys were completed by 95 of 132 round 1 participants (72.0%) for prevention and 95 of 133 (71.4%) participants for treatment outcomes. Consensus meetings agreed four outcomes for the prevention COS: (1) adverse events, (2) development of new comorbidity, (3) health risk behaviour and (4) quality of life; and four for the treatment COS: (1) adherence to treatment, (2) adverse events, (3) out-of-pocket expenditure and (4) quality of life.

**Conclusion:**

Following established guidelines, we developed two COS for trials of interventions for multimorbidity prevention and treatment, specific to adults in LMIC contexts. We recommend their inclusion in future trials to meaningfully advance the field of multimorbidity research in LMICs.

**PROSPERO registration number:**

CRD42020197293.

**WHAT IS ALREADY KNOWN ON THIS TOPIC**

- Although a core outcome set (COS) for the study of multimorbidity has been developed previously, it does not include contributions from low and middle-income countries (LMICs). Given the important differences in disease patterns and healthcare systems between high-income country and LMIC contexts, a fit-for-purpose COS for the study of multimorbidity specific to LMICs is urgently needed.

**WHAT THIS STUDY ADDS**

- Following rigorous guidelines and best practice recommendations for developing COS, we have identified four core outcomes for inclusion in trials of interventions for the prevention and four for the treatment of multimorbidity in adults in LMIC settings.
- The outcomes ‘adverse events’ and ‘quality of life (including Health-related quality of life)’ featured in both prevention and treatment COS. In addition, the prevention COS included ‘development of new comorbidity’ and ‘health risk behaviour’, whereas the treatment COS included ‘adherence to treatment’ and ‘out-of-pocket expenditure’ outcomes.

**HOW THIS STUDY MIGHT AFFECT RESEARCH, PRACTICE OR POLICY**

- These multimorbidity prevention and treatment COS will inform future trials and intervention study designs conducted in LMIC settings by helping promote consistency in outcome selection and reporting.
- COS for multimorbidity interventions that are context-sensitive will likely contribute to reduced research waste, harmonise outcomes to be measured across trials, and advance the field of multimorbidity research in LMIC settings to enhance health outcomes for those living with multimorbidity.

## Introduction

 Multimorbidity, defined as living with two or more long-term health conditions,^1^,^3^ is a growing public health challenge across the world.^4^,^6^ In low and middle-income countries (LMICs), the pooled prevalence of multimorbidity in community settings is estimated to be around 30%.^7^ It is associated with considerable financial burden^8^ and healthcare utilisation that creates strain on often poorly resourced health systems.^9^ In addition, multimorbidity occurs at younger ages in LMICs, reducing quality of life, productivity and life expectancy.^10^

To prevent and improve the treatment of multimorbidity, evidence-based interventions are needed. However, the current heterogeneity of outcomes reported in trials and uncertainty about what should be measured hamper research efforts and limit the ability to compare and synthesise evidence of effectiveness across studies and settings.^11^ Also, the choice of outcomes tends to be driven by researchers’ interests, leading to concerns that the measured outcomes are more important to certain stakeholders, notably researchers and health professionals, not people with lived experience of multimorbidity.^12^ This may be particularly the case in LMICs, where the patient and carer voice in health research and representation in research processes are often limited^13 14^ or can be marginalised due to challenges such as limited health literacy, low socioeconomic status, cultural stigma, and uncertain roles.^15^

A core outcome set (COS) is a minimum set of outcomes (ie, measurements or observations used to capture the effect of interventions^16^) agreed by a range of stakeholders to be the most important for measuring and reporting in *all* studies relating to a specific health condition.^17^ The Core Outcome Measures in Effectiveness Trials (COMET) Initiative has developed rigorous methods for COS identification that are continuously updated.^18 19^ For studies addressing multimorbidity, a COS has been previously developed.^20^ However, it focused only on treatment and did not include prevention outcomes. Importantly, its preparatory work to identify candidate outcomes drew on published research mainly from North America.^21^ Furthermore, the Delphi panel used to achieve consensus on the final COS did not have representation from LMIC contexts. These gaps are important to address, given that both health and economic data pertaining to multimorbidity suggest that prevention may be the best course of action.^22^ In addition, there are marked differences between high-income countries (HIC) and LMIC contexts in populations, healthcare systems, resources, the prevalence and presentation of health conditions and the roles of family members and caregivers.^23 24^ Outcomes identified as important in HICs may not be as relevant in LMIC contexts. Therefore, we aimed to develop two COS for future intervention studies relating to (1) prevention and (2) treatment of multimorbidity among adults residing in LMICs.

## Methods

We followed best practices for COS development, as set out in the COMET guidelines.^16^ We report our steps using the Core Outcome Set-STandards for Reporting (COS-STAR) statement^25^ (online supplemental appendix 1). The COSMOS project is registered with the COMET Initiative (https://www.comet-initiative.org/Studies/Details/1580).

Our COS development involved two main stages: (1) outcome generation stage (identifying a long list of potential outcomes that have been or could be measured in trials) through systematic review and qualitative interviews, followed by (2) an agreement stage on the relative importance of identified outcomes for inclusion in the COS, through Delphi surveys and consensus meetings. Outcomes relevant to the prevention and treatment of multimorbidity were considered separately at each of the stages. The overall study was guided by an expert group, which included global health multimorbidity researchers, clinicians, experts in COS development methods as well as people from LMICs with lived experience of multimorbidity and carer representatives. The main steps of the different stages are described below, and the published protocol provides further details.^26^

### Outcome generation stage

#### Systematic review

We conducted a systematic review with a preregistered protocol to identify outcomes reported in published trials and trial registrations of interventions for the prevention and treatment of multimorbidity in LMICs. Randomised (individual, cluster and cross-over) studies of interventions (pharmacological, non-pharmacological, simple and complex) for multimorbidity in adults (≥18 years) at risk of, or living with multimorbidity, in community, primary care and hospital settings in LMICs were eligible for inclusion. We did not use any weighting of morbidities for study inclusion.

The search strategy was developed by an information specialist (JMW) with inputs from research experts on multimorbidity in LMICs. It included terms for multimorbidity, trial design and terms and names of LMICs, defined according to the 2019 World Bank classification.^27^ We searched 15 electronic databases, including trial registries, and LMIC-specific databases, from 1990 to July 2020 (online supplemental appendix 2). Each record was independently screened by two researchers, first by title and abstract, then by full texts of potentially relevant studies. Any discordance was resolved by discussion or consultation with a third researcher when required. Data on study characteristics, outcomes and outcome measures were extracted from included studies by one researcher, with 10% of extractions cross-verified by a senior researcher. The objective of the review was to compile a list of previously studied outcomes rather than to summarise intervention effect; therefore, study quality was not assessed.^26^

Separate outcome lists were generated for prevention and treatment of multimorbidity, and outcomes were removed or combined based on the following criteria: duplicates, disease-specific (rather than relevant to multimorbidity) or outcome measurement metrics/tools rather than an outcome itself (eg, biochemical measures such as lipid profile, HbA1c, etc, and questionnaires such as Short Form Health Survey (SF-36, SF-12, etc)).

#### Qualitative interviews

To identify outcomes of importance to people with lived experience, qualitative interviews were conducted by enrolling consenting individuals (≥18 years), either living with or caring for someone with multimorbidity. Participants were selected from across a range of LMICs in diverse geographic locations. We used our existing research networks and partnerships to identify in-country research teams with experience of conducting interviews and available to perform data collection. Eligible participants were purposely recruited by these teams to achieve optimal variation according to age (over/under 65 years), sex (male/female) and type of healthcare utilisation (community or primary care/secondary or specialist care).

An information sheet written in plain language was provided to all participants to clarify concepts of outcomes and COS. Informed consent (written or recorded) in the local language was obtained prior to conducting interviews. A semistructured interview guide was used, which was developed in English and translated into the appropriate local languages using standard forward and back translation techniques. The main topics included participants’ experience of living with (or caring for someone living with) multimorbidity and their view on what matters as the result of interventions to prevent or treat and/or care for their conditions. The interview schedule was published as part of the protocol.^26^ Interviews were conducted in-person by qualified interviewers in local languages and either audio-recorded, or if not possible (because of technology limitations or the participant withholding consent), recorded in detailed interviewer notes. Sections of the recordings pertaining to health outcomes were transcribed manually by the local teams and translated into English. Anonymised transcripts were sent to the COSMOS team in York for analysis. Three team members (HK, JRB, RA) reviewed the extracted statements and identified individual multimorbidity prevention and treatment outcomes from them following iterative discussion.

Outcomes identified by the systematic review and interviews were assigned to either prevention or treatment lists or both as appropriate. Lay descriptions were constructed for each outcome and reviewed before finalisation to ensure understanding across all stakeholder groups. Finally, the prevention and treatment outcomes were categorised for presentation to the Delphi panels, using Dodd’s outcome taxonomy comprising 38 categories across five core areas, namely death, physiological/clinical, life impact, resource use and adverse events.^28^

### Agreement stage

#### Delphi surveys

We conducted two rounds of online Delphi surveys to reach consensus on the importance of each outcome identified by the outcome generation stage; separate surveys were conducted for prevention and treatment outcomes. Participants were purposively sought from across four stakeholder groups, namely (1) people living with multimorbidity and their caregivers, (2) healthcare professionals, (3) policymakers and (4) multimorbidity researchers. The identification and recruitment of participants used multiple strategies such as broadcasting through a project Twitter account, patient and public involvement groups and COSMOS team networks (including other global health research groups, professional societies, non-government organisations relevant to multimorbidity and government ministries). Additional strategies to recruit healthcare professionals and multimorbidity researchers included personalised emails sent to corresponding authors of studies included in our systematic review and flyers posted in partner research organisations.

We used the DelphiManager V.5.0 platform, developed and maintained by the COMET Initiative (University of Liverpool),^18^ to administer all surveys. The order of presenting outcome domains (based on Dodd’s taxonomy) was randomised to reduce bias. A lay description for each outcome was provided. Survey participants were asked to score the importance of each outcome for inclusion in the prevention and treatment COS, without considering its feasibility or measurability. For scoring, the Grading of Recommendations Assessment, Development and Evaluations (GRADE) 9-point Likert scale was used, with the following categories^16^: ‘not important’ (scores 1–3), ‘important but not critical’^4^,^6^ and ‘critical for inclusion’.^7^,^9^ There was also an ‘Unable to Score’ response option (for participants who felt they did not have the specific knowledge or understanding to score on a particular outcome) as well as the opportunity to suggest additional outcomes. For Delphi round 1, for each outcome, we determined the proportion of scores in each of the GRADE categories, both overall and for each stakeholder group. All additional outcomes suggested by survey participants were reviewed for duplication and relevance by the research team, and those eligible (new distinct outcomes relevant to multimorbidity studies) were included in the Delphi round 2 surveys.

For Delphi round 2, participants received their own round 1 scores as well as the summary scores (overall and for each stakeholder group), with visual representation using histograms of the proportion of scores in each GRADE category. Participants were asked to rescore the importance of each outcome using the same 9-point Likert scale and to provide free-text reasons for any changes. Reminder emails were sent for both Delphi rounds until a minimum acceptable level of participation was achieved (70% overall, as advised by COS experts). Ratings from round 2 were analysed and summarised as for round 1 under the GRADE categories. Outcomes were then grouped according to the consensus definitions recommended by COMET (see table 1).^11^ To aid understanding of the findings and indicate clearly where there was consensus across stakeholder groups (or its absence), outcomes were presented in colour-coded tables (table 1).

**Table 1:** Criteria for categorisation of outcomes in the Delphi surveys

Green	Outcomes scored ‘critical for inclusion’ (7**–**9) by>70% of respondents in all four stakeholder groups.
Purple	Outcomes scored ‘critical for inclusion’ (7**–**9) by>70% of respondents in three of four stakeholder groups.
Blue	Outcomes scored ‘critical for inclusion’ (7**–**9) by>70% of respondents in two of four stakeholder groups.
Yellow	Outcomes scored ‘critical for inclusion’ (7**–**9) by>70% of respondents in one of four stakeholder group.
Red	Outcomes scored ‘critical for inclusion’ (7**–**9) by<70% of the respondents in all four stakeholder groups.

### Consensus meetings

All Delphi participants were sent electronic invitations for the consensus meetings. A modified nominal group technique was used to discuss findings from the Delphi surveys and to develop agreements on critical outcomes for inclusion in the COS.^16 29 30^ Separate meetings were held for prevention and treatment outcomes, using the Zoom online platform^31^ to maximise participation from multiple countries. Two premeeting sessions were held to orientate attendees to the purpose of the consensus meetings, scope of the COS and the use of Zoom. In addition, an information pack describing the processes followed in the study and presenting results from the Delphi surveys using colour-coded tables (as described above) were sent to all participants before the meetings. Those participants unable to attend the virtual meetings were invited to send in their views by email.

At the start of each meeting, we reminded participants of the aim (ie, developing consensus on the inclusion of outcomes in the COS) and outlined the meeting structure and process to ensure inclusive discussions. Meetings were facilitated by experts with extensive experience in COS development (JK, LR). Results from the Delphi surveys were presented. Outcomes scored as ‘critical for inclusion’ by >70% of Delphi respondents in all four stakeholder groups (colour-coded green, see table 1) were included in the COS if they meet the consensus meeting threshold of ≥80% voting for inclusion; otherwise, they underwent further discussion. Outcomes scored as ‘critical for inclusion’ by >70% of Delphi respondents in only one or no stakeholder groups (colour-coded yellow/red) were excluded without further discussion unless nominated to be ‘saved’ and supported by voting above a threshold of ≥80% by meeting participants. All outcomes scored as ‘critical for inclusion’ by >70% of Delphi respondents in two or three stakeholder groups (colour-coded blue/purple) were discussed further. Views shared by email by individuals unable to attend meetings were also fed into the meeting. Iterative rounds of whole-group and small-group discussions, facilitated by Google Jamboard, were used to categorise the outcomes for discussion into ‘critical’ ‘good to include’ and ‘not important’. Discussions were followed by voting to include or exclude outcomes in the COS.

Following the consensus meetings, participants were emailed for a further vote on any outcomes for which consensus was not reached during the meetings, and for feedback on the wording and descriptions of outcomes voted for inclusion. The two final COS for prevention and treatment were compiled and sent to all consensus meeting participants for final endorsement.

### Patient and public involvement

Four members of the steering committee overseeing the study were people living with multimorbidity and their caregivers. The study also benefited from advice from the NIHR IMPACT in South Asia Group (https://www.impactsouthasia.com/impact-group/) Community Advisory Panels and from the NCD Alliance (https://ncdalliance.org/), a civil society network, advocating for people with non-communicable diseases.

## Results

### Outcome generation stage

**Figure 1:**
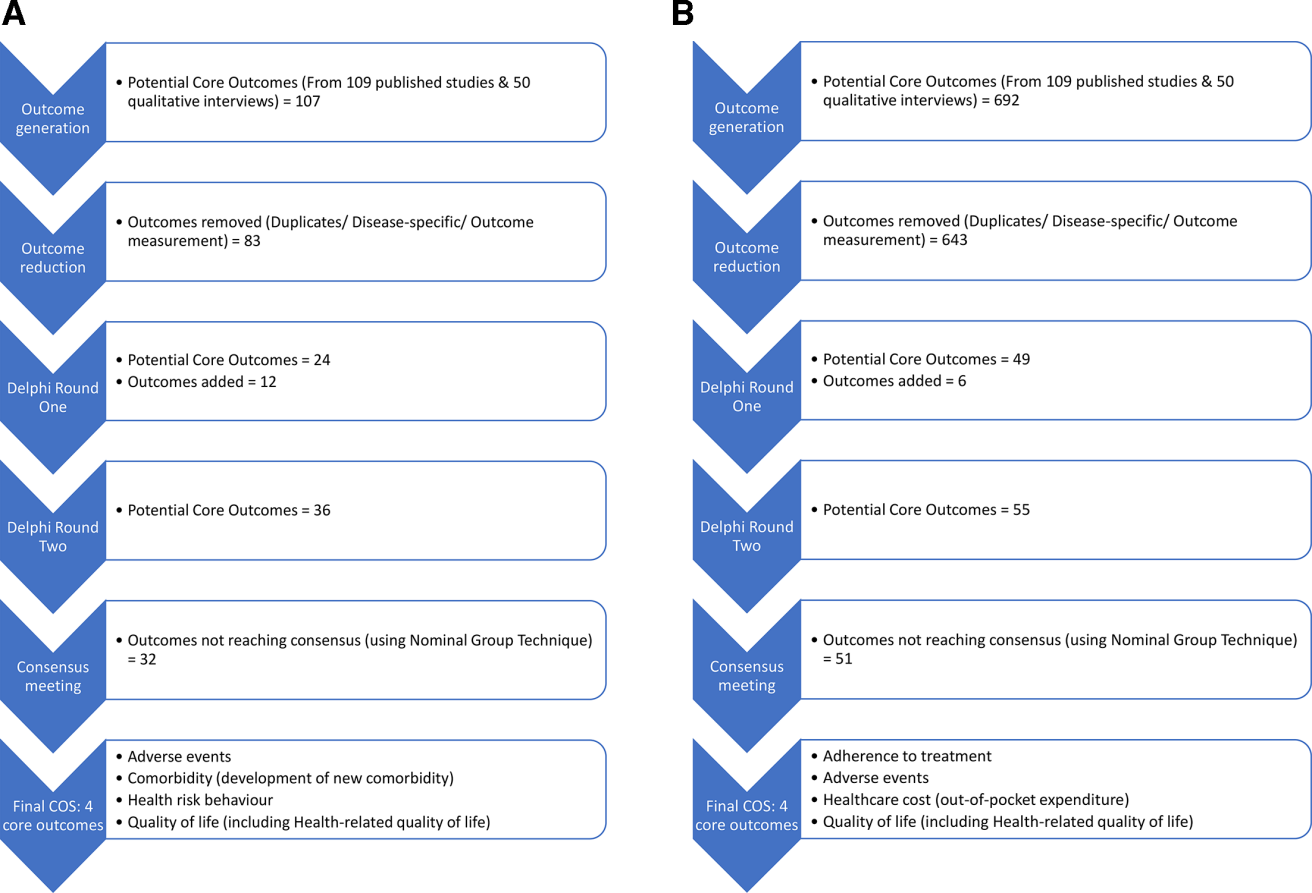
(A) Development of COS for trials of interventions to prevent multimorbidity in LMICs. (B) Development of COS for trials of interventions to treat multimorbidity in LMICs. COS, core outcome sets; LMICs, low and middle-income countries.

Figure 1A,B shows the steps of outcome generation related to the prevention and treatment of multimorbidity, respectively.

#### Systematic review

Our searches yielded 17 267 records, with 16 949 remaining after removing duplication publications (online supplemental appendix 2). Following title and abstract screening, 16 705 records were excluded, and the remaining 243 papers were obtained. Full-text screening resulted in the exclusion of a further 134 records (online supplemental appendix 2). The remaining 109 randomised intervention studies on the prevention and treatment of multimorbidity conducted in at least 25 LMICs were included.

From these papers, 92 prevention and 236 treatment outcomes were extracted and reduced to 19 prevention and 38 treatment outcomes after removing duplicate outcomes, disease-specific outcomes and outcome measurement metrics.

#### Qualitative interviews

The interviewees included five participants from each of the following 10 countries: Afghanistan, Burkina Faso (low-income), Bangladesh, Ghana, Nepal, Nigeria, Pakistan (lower middle income), Mexico, Peru and Suriname (upper-middle income), totalling 50 interviewees. They comprised 37 people living with multimorbidity and 13 family caregivers. The distribution of sociodemographic characteristics was as follows: sex (46% male and 54% female), age (80% under 65 and 20% 65+ years) and type of healthcare utilisation (34% community/primary care and 66% secondary/specialist care). Participants reported having from two to five coexisting conditions, including tuberculosis, asthma, hypertension, diabetes, cardiovascular disease, HIV, cancer, stroke, mental health disorders and others.

The interviews generated five further outcomes for prevention and 11 for treatment of multimorbidity (see online supplemental appendix 4, eg, coding of qualitative data). Combining these outcome lists with the corresponding lists generated from the systematic review resulted in 24 outcomes for prevention and 49 for treatment of multimorbidity, which were classified according to Dodd’s taxonomy and presented in the Delphi round 1 surveys (online supplemental appendix 5).

### Agreement stage

Table 2 summarises the characteristics of participants in the Delphi surveys and consensus meetings; table 3 shows the outcomes scored as critical for inclusion at each of the agreement stages.

**Table 2:** Characteristics of participants in Delphi surveys and consensus meetings

Name of survey/meeting	Stakeholder group, n (%)	Age group, n (%)	Female, n (%)	Region, n (%)
Delphi round 1, prevention survey (N=132)	People living with multimorbidity/caregivers=29 (22.0) Healthcare professionals=27 (20.4) Policymakers=12 (9.1) Multimorbidity researchers=64 (48.5)	18–24=4 (3.0) 25–34=34 (25.7) 35–44=34 (25.7) 45–54=26 (19.7) 55–64=24 (18.2) 65+=10 (7.6)	68 (51.5)	Low income=6 (4.5) Lower middle income=74 (56.1) Upper middle income=15 (11.4) High income=37 (28.0)
Delphi round 1, treatment survey (N=133)	People living with multimorbidity/caregivers=30 (22.5) Healthcare professionals=24 (18.0) Policymakers=13 (9.8) Multimorbidity researchers=66 (49.6)	18–24=3 (2.2) 25–34=36 (27.0) 35–44=35 (26.3) 45–54=26 (19.5) 55–64=22 (16.5) 65+=11 (8.4)	70 (52.6)	Low income=4 (3.0) Lower middle income=78 (58.6) Upper middle income=17 (12.8) High income=34 (25.6)
Delphi round 2, prevention survey (N=95)	People living with multimorbidity/caregivers=24 (25.3) Healthcare professionals=14 (14.7) Policymakers=8 (8.4) Multimorbidity researchers=49 (51.6)	NA	49 (51.6)	Low income=5 (5.3) Lower middle income=45 (47.4) Upper middle income=10 (10.5) High income=35 (36.8)
Delphi round 2, treatment survey (N=95)	People living with multimorbidity/caregivers=22 (23.1) Healthcare professionals=16 (16.8) Policymakers=10 (10.5) Multimorbidity researchers=47 (49.5)	NA	51 (53.7)	Low income=5 (5.3) Lower middle income=48 (50.5) Upper middle income=11 (11.6) High income=31 (32.6)
Consensus meeting, prevention (N=19)	People living with multimorbidity/caregivers=3 (15.8) Healthcare professionals=4 (21.0) Policymakers=1 (5.3) Multimorbidity researchers=11 (57.9)	NA	10 (52.6)	Low income=1 (5.3) Lower middle income=11 (57.9) Upper middle income=1 (5.3) High income=6 (31.6)
Consensus meeting, treatment (N=14)	People living with multimorbidity/caregivers=1 (7.1) Healthcare professionals=4 (28.6) Policymakers=1 (7.1) Multimorbidity researchers=8 (57.1)	NA	7 (50.0%)	Low income=0 (0.0) Lower middle income=6 (42.8) Upper middle income=4 (28.6) High income=4 (28.6)

**Table 3:** Critical outcomes selected in Delphi surveys and consensus meetings

Delphi round 1	Delphi round 2	Delphi results	Consensus
**Prevention outcomes**			
**‘Critical for inclusion’ by≥70% of all participants**	**Voted ‘critical for inclusion’ (7-9) by≥70% of all participants**	**‘Critical for inclusion’ by≥70% in 4 (green), 3 (purple) and 2 (blue) stakeholder groups**	**Prevention COS**
Adherence to treatment Adverse events Cardiovascular event Cardiovascular risk Cognitive function Comorbidity Cost-effectiveness Death Health-related quality of life Obesity Organ damage Prevention of hypertension Psychological well-being Quality of life Timely screening	Adherence to treatment Adverse events Cardiovascular event Cardiovascular risk Chronic disease self-management* Comorbidity Cost-effectiveness Death Health risk behaviour* Health-related quality of life Obesity Organ damage Prevention of hypertension Psychological well-being Quality of life Timely screening	Green-coded outcomes: Adverse events Cardiovascular event Chronic disease self-management* Comorbidity Prevention of hypertension Quality of life Purple-coded outcomes: Cost-effectiveness Death Obesity Organ damage Pain Psychological well-being Timely screening Blue-coded outcomes: Adherence to treatment Cardiovascular risk Cognitive function Diet Early detection* Health risk behaviour* Health-related quality of life Treatment satisfaction	Adverse events Comorbidity (development of new comorbidity) Health risk behaviour* Quality of life (including Health-related quality of life)
**Other outcomes**	**Other outcomes**	**‘Critical for inclusion’ by≥70% in 1 (yellow), or none (red) of the stakeholder groups**	**Not in COS, but suggested as additional outcome**
Diet Exercise tolerance Fatigue Health literacy Healthcare use Pain Reduced medication Treatment satisfaction Weight	Carer burden* Cognitive function Diet Early detection* Exercise tolerance Fatigue Functioning/ADL* Health anxiety* Health literacy Health-seeking behaviour* Healthcare use Income* Loneliness* Pain Perceived health* Reduced medication Self-efficacy* Social functionality* Treatment satisfaction Weight	Yellow-coded outcomes: Carer burden* Health literacy Self-efficacy* Weight Red-coded outcomes: Exercise tolerance Fatigue Functioning/ADL* Health anxiety* Health-seeking behaviour* Healthcare use Income* Loneliness* Perceived health* Reduced medication Social functionality*	Healthcare use (including cost-effectiveness)
**Treatment outcomes**			
**‘Critical for inclusion’ by≥70% of all participants**	**‘Critical for inclusion’ by≥70% of all participants**	**‘Critical for Inclusion’ by ≥70% in 4 (green), 3 (purple), and 2 (blue) stakeholder groups**	**Treatment COS**
Adherence to treatment Adverse events Cardiovascular event Cognitive function Comorbidity Cost-effectiveness Death Healthcare access Healthcare cost Healthcare quality Health-related quality of life Increase in symptoms Psychological well-being Quality of life Treatment satisfaction	Adherence to treatment Adverse events Cardiac event risk Cardiovascular event Cognitive function Comorbidity Cost-effectiveness Death Healthcare access Healthcare cost Healthcare quality Health-related quality of life Hospital admission Illness under control Psychological well-being Quality of life Treatment satisfaction	Green-coded outcomes: Adherence to treatment Death Healthcare access Healthcare cost Healthcare quality Purple-coded outcomes: Adverse events Cardiovascular event Comorbidity Cost-effectiveness Health-related quality of life Healthcare staff communication Increase in symptoms Pain Psychological well-being Quality of life Treatment burden† Treatment satisfaction	Adherence to treatment Adverse events Healthcare cost (out-of-pocket cost of treatment) Quality of life (including Health-related quality of life)
		Blue-coded outcomes: Cardiovascular risk Cognitive function Continuity of care† Falls risk Health risk behaviour Healthcare use Hospital admission Hypertension Illness under control Obesity Perceived health Physical activity	
**Other outcomes**	**Other outcomes**	**‘Critical for inclusion’ by ≥70% in 1 (yellow), or none (red) of the stakeholder groups**	**Not in COS, but suggested as additional outcome**
Acceptance of illness Aggression Agitation Appetite Balance Cardiac event risk Carer burden Diet Domestic violence Emotional regulation Falls risk Fatigue Functioning/ADL Health anxiety Health literacy Health risk behaviour Healthcare staff communication Healthcare use Hospital admission Hypertension Illness resolution Illness stigma Illness under control Income Loneliness Nausea Obesity Pain Perceived health Physical activity Reduced medication Self-management Sleep quality Weight	Acceptance of illness Aggression Agitation Appetite Balance Carer burden Continuity of care† Diet Domestic violence Emotional regulation Falls risk Fatigue Frailty† Functioning/ADL Health anxiety Health literacy Health risk behaviour Healthcare staff communication Healthcare use Hypertension Illness resolution Illness stigma Income Increase in symptoms Loneliness Nausea Obesity Pain Perceived health Physical activity Polypharmacy† Reduced medication Self-esteem† Self-management Sleep quality Social functionality† Treatment burden† Weight	Yellow-coded outcomes: Functioning/ADL Illness resolution Income Polypharmacy† Self-management Sleep quality Social functionality† Red-coded outcomes: Acceptance of illness Aggression Agitation Appetite Balance Carer burden Diet Domestic violence Emotional regulation Fatigue Frailty† Health anxiety Health literacy Illness stigma Loneliness Nausea Reduced medication Self-esteem† Weight	N/A

ADL - Activities of Daily Living.

* Additional prevention outcomes suggested during Delphi round 1.

† Additional treatment outcomes suggested during Delphi round 1 (see online supplemental appendix 5 for help text).

COS, core outcome sets.

#### Delphi surveys

The Delphi round 1 prevention and treatment surveys were completed by 132 and 133 participants, respectively, with 127 completing both. The distribution of stakeholders was similar in both groups, with multimorbidity researchers making up almost half the sample, followed by people living with multimorbidity/caregivers (~22%), healthcare professionals (18%–20%) and policymakers (<10%). Over half of the participants in both groups were 25–44 years old and women. The largest geographical representation in both groups was from lower LMICs (56%–58%), followed by high income (25%–28%), upper middle income (~12%) and low-income countries (<5%). The Delphi round 2 surveys were completed by 95 participants, for prevention (72.0% of round 1) and treatment outcomes (71.4% of round 1). By stakeholder groups, round 2 completions were >70% of round 1 participants for all groups, except healthcare professionals (66.7% in prevention and treatment surveys) and policymakers (51.8% in prevention survey).

Of the outcomes presented in Delphi round 1, 15 (of 24) prevention and 15 (of 49) treatment outcomes were rated as ‘critical for inclusion’ (scores 7–9) by ≥70% of all participants (table 3). Thirty-eight additional outcomes were proposed for prevention with 12 of them included in Delphi round 2 after reviewing for duplication, and relevance to multimorbidity. For treatment, 6 of 24 proposed additional outcomes were taken forward to Delphi round 2. Overall, 36 prevention (24 generated from the review and interviews and 12 additional suggestions by Delphi round 1 respondents) and 55 treatment outcomes (49 generated and 6 additional suggestions) were presented for rating in the Delphi round 2 surveys.

In the Delphi round 2 surveys, 16 (of 36) prevention and 17 (of 55) treatment outcomes were rated as ‘critical for inclusion’ (scores 7–9) by ≥70% of all participants (table 3). Categorising by stakeholder groups, in the prevention list, six outcomes were coded green (‘critical for inclusion’ by >70% in all stakeholder groups), 15 were coded blue/purple (‘critical for inclusion’ by >70% in any three or two stakeholder groups), and 15 were coded yellow/red (‘critical for inclusion’ by >70% in one or none of the stakeholder groups); the treatment list included 5 (green), 31 (blue/purple), and 19 (yellow/red) outcomes, following the same categorisation (table 3).

#### Consensus meetings

##### Prevention

The consensus meeting for prevention had 17 in-meeting and two email participants (table 2), including 11 (57.9%) multimorbidity researchers, three people living with multimorbidity/caregivers (15.8%), four healthcare professionals (21.0%) and one policymaker (5.3%). Following the nominal group technique discussions, 32 of the 36 outcomes were excluded; there was consensus on the four outcomes presented below for inclusion in the prevention COS (table 3).

‘Comorbidity’ and ‘quality of life’ (both green-coded outcomes) received 93% of votes in the consensus meeting, but both outcomes were recommended for further discussion regarding their wording. Following these discussions, for the prevention COS, it was agreed ‘comorbidity’ referred to the prevention of development of a new illness alongside the existing health condition being examined in a trial; the wording of this outcome was, therefore, amended as ‘comorbidity (development of new comorbidity)’. Similarly, ‘quality of life’ was reworded as ‘quality of life (including health-related quality of life)’, to reflect the consensus that the two outcomes should be combined (with researchers free to choose the most appropriate measure for their trial).

‘Adverse events’ (green-coded) did not initially reach the voting threshold of ≥80%, with those opposing its inclusion suggesting that measuring such events would be common practice across studies. However, it was included following further discussion and consensus among meeting participants, who considered it incorporated a range of negative outcomes for research teams to decide as appropriate for the multimorbidity prevention intervention being implemented.

The outcome ‘health risk behaviours’ (blue-coded), proposed for the prevention COS during the Delphi round 1 survey, was similarly included following consensus discussions. The term was understood to include the range of behaviours considered to be risk factors for chronic conditions such as tobacco use, physical inactivity and unhealthy diet.

The outcome ‘Healthcare use’ (including cost-effectiveness) generated extensive debate. While it was considered very important, it was argued that it might not be relevant to all trials. ‘Healthcare use’ did not reach the voting threshold for inclusion in the COS for this reason, but the meeting consensus was that it should be recommended as an important outcome to consider in relevant trials. Other outcomes such as ‘Cardiovascular event’ and ‘Prevention of hypertension’, although green coded following the Delphi surveys, did not reach the consensus threshold as participants considered they were too condition-specific.

##### Treatment

The treatment consensus meeting comprised 12 in-meeting and two email participants (table 2), including 8 (57.1%) multimorbidity researchers, four healthcare professionals (28.6%), one person living with multimorbidity/caregiver (7.1%) and one policymaker (7.1%). There was consensus on the following four outcomes as critical for inclusion (table 3).

‘Adherence to treatment’ and ‘healthcare costs’ (both green-coded outcomes) received 90% and 100% of votes, respectively; additional clarification was added to the latter, stipulating it was specifically ‘out-of-pocket expenditure’ that was considered critical in LMIC studies, and as such this should be specified in the COS. The outcomes ‘adverse events’ and ‘quality of life’ (including Health-related quality of life) (both purple-coded) were included after further discussion and consensus. While it was agreed that death should be reported as an adverse event where relevant, the outcome ‘Death’ or ‘Mortality’ did not reach the threshold for inclusion separately (60% votes). Similarly, ‘Healthcare access’ and ‘Healthcare quality’ (both green-coded outcomes) did not reach the threshold for inclusion, as participants perceived them to be mediators rather than outcomes.

## Discussion

The COSMOS study followed rigorous participatory methods as recommended by COMET with representation from diverse geographies and stakeholder groups to develop two COS for use in future trials of interventions to prevent and to treat multimorbidity among adults living in LMICs. Our COS did not extend to paediatric populations, as we anticipated different outcomes and needs.^32 33^ The two COS included four outcomes each, with ‘adverse events’ and ‘quality of life (including health-related quality of life)’ featured in both sets. In addition, the prevention COS included ‘development of new comorbidity’ and ‘health risk behaviour’, whereas the treatment COS included ‘adherence to treatment’ and ‘out-of-pocket expenditure’ outcomes.

A previously developed COS for multimorbidity (COSmm)^20^ with inputs from a systematic review of studies^21^ and an expert panel, both solely from HICs, also included ‘Health-related quality of life’ among their highest scoring outcomes. Our consensus panels voted to combine this outcome with the broader ‘quality of life’ both in the prevention and treatment COS. This similarity between COSmm and our results suggests that the outcome ‘quality of life’ may be relevant to multiple stakeholders and well-suited across diverse contexts to capture the impacts of living with multimorbidity. Nonetheless, further work on differentiating these constructs and their operationalisation will be necessary to translate this finding into actionable research and clinical practice.^34 35^ The inclusion of adherence to treatment’ and ‘healthcare costs’ is further similarity between COSmm and our treatment COS. However, while costs are only presented as a broad health systems outcome in COSmm, we specify its scope as covering out-of-pocket treatment costs to people living with multimorbidity, given that this can be an important source of catastrophic health expenditures and impoverishment in many LMICs.^13 36 37^

‘Healthcare use’ was included in COSmm but did not reach a consensus for inclusion in our LMIC COS. This likely reflects differences across LMICs in the use of healthcare services.^38^ Additionally, the outcome may be severely limited in some LMICs due to lack of access to services.^39^ Nevertheless, it was considered an important outcome, which should be included (with or without cost-effectiveness) in some prevention studies, where appropriate. We thought it particularly important to develop a separate COS for the prevention of multimorbidity, given that the targets for prevention and treatment interventions are often different. Furthermore, as non-communicable diseases (with amenable risk factors) form a large proportion of the multimorbidity burden, a COS for prevention trials that will help build the evidence base is critical. With clear opportunities for implementing prevention strategies targeting risk factors,^40^ it is noteworthy that ‘health risk behaviour’ has been included in our prevention COS. This outcome was added following the Delphi round 1 suggestions, indicating that it was not captured in previous research on multimorbidity.

Currently, most COS reflect priorities from HIC perspectives only, with very few including participants from LMICs and even fewer initiated in LMICs.^13 41 42^ Given that there are important differences in populations, disease patterns and healthcare systems between HIC and LMIC contexts,^23 24^ our two COS for intervention studies to prevent and treat multimorbidity specifically in LMICs are likely to be more context-relevant, with greater applicability and adoptability in these settings.^43^ Another advantage of the COS will be more consistent and aligned outcome reporting in future multimorbidity trials, leading to systematic reviews that are more meaningful, as like-for-like outcomes can be combined in meta-analyses.

Having agreed COS for LMIC multimorbidity trials, further work is needed to review the evidence base and develop consensus on validated metrics or tools, which should be used to capture these outcomes. This was beyond the scope of the current study, but we have collated from our systematic review the measures and tools used to assess the six outcomes included in the two multimorbidity COS (see online supplemental appendix 6). Also, the large difference in the number of potential outcomes identified for prevention trials (N=107) and treatment trials (N=692) illustrates the need for more research on (and implementation of) preventive interventions.

The strengths of our study include adherence to the recommended COMET guidelines^16^ at both the outcome generation and agreement stages. We used a combination of rigorously conducted approaches (systematic review and qualitative interviews) to generate the initial lists of prevention and treatment outcomes, and multistage consensus building exercises involving a wide range of stakeholders across backgrounds, professions and countries.

There are three key limitations to consider. First, unlike the interviews conducted in local languages, the Delphi surveys were administered in English, using the DelphiManager online platform, thereby limiting participation to individuals who could read or speak English and had a degree of confidence in using online tools. To mitigate the impact of this, support was provided by in-country research partners, but this was challenging to do consistently for Delphi round 2, which led to higher than anticipated attrition. Nonetheless, the study achieved a satisfactory response rate in the round 2 surveys (>70.0% of round 1 participants for both prevention and treatment rounds), with representation from across 33 countries, which may not have been possible without using online tools. The countries represented were mainly from South Asia and Europe, likely reflecting our research team networks.

Another limitation was that multimorbidity researchers were the largest stakeholder group in the agreement stages, with the risk that the consensus and final COS might largely reflect their views. Policymakers, on the other hand, had the least representation. However, our approach ensured that views were included from all four stakeholder groups at all consensus-building stages (table 2). Delphi survey responses were summarised by stakeholder group, with agreement across groups being a key consideration in identifying outcomes as important. The selection of outcomes for the COS in consensus meetings also took account of their importance for all stakeholder groups, with particular consideration to the perspectives of those with lived experience.

Finally, methods for COS development are evolving.^44^ While our approach adheres to the currently recommended steps and represents an advance over the previous COS for multimorbidity, the evidence base for developing consensus is limited^45^ (eg, on the optimum way to present results in Delphi surveys, or to conduct discussions and achieve equitable, inclusive ranking or voting on outcomes). We further acknowledge that continued efforts are needed to understand the uptake and impact of COS, as demonstrated in other areas of health.^46^

In addition, the definition of an intervention to prevent and/or treat multimorbidity might itself need further development.^47^ Repeatedly identified issues in the management of multimorbidity are the lack of integrated care and inadequate considerations of cross-treatment interactions, complications and consequences.^48^ Interventions that consider these issues might be ones which have a planned positive impact on one or more conditions, while considerations are undertaken to minimise, reduce or avoid negative impacts from the presence of multimorbidity. Future efforts may be needed to include this broader scope.

## Conclusion

In conclusion, the COSMOS study has developed two COS specifically for LMICs, to include in all intervention studies focusing on the prevention and treatment of multimorbidity. The two COS comprise four outcomes each, carefully selected using recommended standards, and therefore likely to be relevant and meaningful to a wide range of LMIC stakeholders, including people living with multimorbidity, their caregivers, multimorbidity researchers, healthcare professionals and policymakers. Future research should identify and develop consensus on validated measures to assess these outcomes. Uptake of COS in future trials will promote consistency in outcome selection and reporting and thereby ensure the comparability of effectiveness across different studies on multimorbidity in LMICs.

## Supplementary material

10.1136/bmjgh-2024-015120 online supplemental appendix 1

## Data Availability

All data relevant to the study are included in the article or uploaded as supplementary information.
