# Supplementary material for: Core outcome sets for trials of interventions to prevent and to treat multimorbidity in adults in low and middle-income countries: the COSMOS study
Source: BMJ Glob Health. 2024 Aug 19;9(8):e015120. doi: 10.1136/bmjgh-2024-015120 (PMC11340216; doi:10.1136/bmjgh-2024-015120)
Supplement: online supplemental appendix 1 [file bmjgh-9-8-s001.pdf]

Core outcome sets for trials of interventions to prevent and to treat multimorbidity in adults in low- and middle-income countries: the COSMOS study.

APPENDICES

Appendix 1: The COS-STAR Statement Checklist

SECTION/TOPIC	ITEM	CHECKLIST ITEM	REPORTED ON PAGE NUMBER
TITLE/ABSTRACT			
Title	1a	Identify in the title that the paper reports the development of a COS	1
Abstract	1b	Provide a structured summary	1-2
INTRODUCTION			
Background and Objectives	2a	Describe the background and explain the rationale for developing the COS.	4
	2b	Describe the specific objectives with reference to developing a COS.	4
Scope	3a	Describe the health condition(s) and population(s) covered by the COS.	4
	3b	Describe the intervention(s) covered by the COS.	4
	3c	Describe the setting(s) in which the COS is to be applied.	4
METHODS			
Protocol/Registry Entry	4	Indicate where the COS development protocol can be accessed, if available, and/or the study registration details.	4
Participants	5	Describe the rationale for stakeholder groups involved in the COS development process, eligibility criteria for participants from each group, and a description of how the individuals involved were identified.	5-7, in different stages

Information Sources	6a	Describe the information sources used to identify an initial list of outcomes.	5, outcome generation stage
	6b	Describe how outcomes were dropped/combined, with reasons (if applicable).	5, also figures 1a and 1b
Consensus Process	7	Describe how the consensus process was undertaken.	6-7, agreement stage
Outcome Scoring	8	Describe how outcomes were scored and how scores were summarised.	6-7, also Table 1
Consensus Definition	9a	Describe the consensus definition.	7
	9b	Describe the procedure for determining how outcomes were included or excluded from consideration during the consensus process.	7
Ethics and Consent	10	Provide a statement regarding the ethics and consent issues for the study.	7
RESULTS			
Protocol Deviations	11	Describe any changes from the protocol (if applicable), with reasons, and describe what impact these changes have on the results.	11, discussion (not major)
Participants	12	Present data on the number and relevant characteristics of the people involved at all stages of COS development.	8-10, in different stages
Outcomes	13a	List all outcomes considered at the start of the consensus process.	Table 3
	13b	Describe any new outcomes introduced and any outcomes dropped, with reasons, during the consensus process.	9, also Table 3
COS	14	List the outcomes in the final COS.	1 (Abstract), 10 (Results), also Table 3
DISCUSSION			
Limitations	15	Discuss any limitations in the COS development process.	11-12
Conclusions	16	Provide an interpretation of the final COS in the context of other evidence, and implications for future research.	12
OTHER INFORMATION			
Funding	17	Describe sources of funding/role of funders.	21

Conflicts of Interest	18	Describe any conflicts of interest within the study team and how these were managed.	21

From: Kirkham JJ, Gorst S, Altman DG, Blazeby JM, Clarke M, Devane D, et al. (2016) Core Outcome Set–STAndards for Reporting: The COS-STAR Statement. PLoS Med 13(10): e1002148. https://doi.org/10.1371/journal.pmed.1002148

Appendix 2: Appendices related to systematic review.

2.1. Databases searched and example search strategy.

1. MEDLINE (Ovid) (see search strategy below)
2. Embase (Ovid)
3. PsycINFO (Ovid)
4. Cochrane Central Register of Controlled Trials (CENTRAL)
5. CINAHL (EBSCOhost)
6. Global Health (Ovid)
7. PakMediNet (https://www.pakmedinet.com/)
8. Global Index Medicus (WHO) (ISMEAR, LILACS, IMEMR, WRIM).
9. Cochrane Database of Systematic Reviews (CDSR)
10. The Database of Abstracts of Reviews of Effects (DARE)
11. PROSPERO (https://www.crd.york.ac.uk/prospero/)
12. Joanna Briggs
13. ClinicalTrials.gov
14. ISRCTN registry (www.isrctn.com)
15. The International Clinical Trials Registry Platform (http://apps.who.int/trialsearch).

Database: Ovid MEDLINE(R) ALL <1946 to July 17, 2020>

Search Strategy:

----

1 Comorbidity/ (107657)

2 (comorbid$ or co-morbid$).ti,ab,kf. (181237)

3 (multimorbid$ or multi-morbid$).ti,ab,kf. (5739)

4 (multidisease$ or multi-disease$ or (multiple adj (ill$ or disease$ or condition$ or syndrome$ or disorder$))).ti,ab,kf. (5517)

5 ((cooccur$ or co-occur$ or coexist$ or co-exist$ or multipl$) adj3 (disease$ or ill$ or care or condition$ or disorder$ or health$ or syndrome$)).ti,ab,kf. (76531)

6 1 or 2 or 3 or 4 or 5 (319021)

7 Cooperative Behavior/ (43408)

8 Interdisciplinary Communication/ (17155)

9 Interprofessional Relations/ (51072)

10 Intersectoral Collaboration/ (1958)

11 Patient Care Team/ (65264)

12 (collaborat$ adj4 care).ti,ab,kf. (8703)

13 ((integrated or interdisciplinary or inter-disciplinary or interprofessional or inter-professional or intersectoral or inter-sectoral or multidisciplnary or multi-disciplinary) adj1 (care or management or practice or treatment)).ti,ab,kf. (12540)

14 7 or 8 or 9 or 10 or 11 or 12 or 13 (168849)

15 6 or 14 (482945)

16 (afghanistan or albania or algeria or american samoa or angola or "antigua and barbuda" or antigua or barbuda or argentina or armenia or armenian or aruba or azerbaijan or bahrain or bangladesh or barbados or republic of belarus or belarus or byelarus or belorussia or byelorussian or belize or british honduras or benin or dahomey or bhutan or bolivia or "bosnia and herzegovina" or bosnia or herzegovina or botswana or bechuanaland or brazil or brasil or bulgaria or burkina faso or burkina fasso or upper volta or burundi or urundi or cabo verde or cape verde or cambodia or kampuchea or khmer republic or cameroon or cameron or cameroun or central african republic or ubangi shari or chad or chile or china or colombia or comoros or comoro islands or iles comores or mayotte or democratic republic of the congo or democratic republic congo or congo or zaire or costa rica or "cote d’ivoire" or "cote d’ ivoire" or cote divoire or cote d ivoire or ivory coast or croatia or cuba or cyprus or czech republic or czechoslovakia or djibouti or french somaliland or dominica or dominican republic or ecuador or egypt or united arab republic or el salvador or equatorial guinea or spanish guinea or eritrea or estonia or eswatini or swaziland or ethiopia or fiji or gabon or gabonese republic or gambia or "georgia (republic)" or georgian or ghana or gold coast or gibraltar or greece or grenada or guam or guatemala or guinea or guinea bissau or guyana or british guiana or haiti or hispaniola or honduras or hungary or india or indonesia or timor or iran or iraq or isle of man or jamaica or jordan or kazakhstan or kazakh or kenya or "democratic people’s republic of korea" or republic of korea or north korea or south korea or korea or kosovo or kyrgyzstan or kirghizia or kirgizstan or kyrgyz republic or kirghiz or laos or lao pdr or "lao people's democratic republic" or latvia or lebanon or lebanese republic or lesotho or basutoland or liberia or libya or libyan arab jamahiriya or lithuania or macau or macao or "macedonia (republic)" or macedonia or madagascar or malagasy republic or malawi or nyasaland or malaysia or malay federation or malaya federation or maldives or indian ocean islands or indian ocean or mali or malta or micronesia or federated states of micronesia or kiribati or marshall islands or nauru or northern mariana islands or palau or tuvalu or mauritania or mauritius or mexico or moldova or moldovian or mongolia or montenegro or morocco or ifni or mozambique or portuguese east africa or myanmar or burma or namibia or nepal or netherlands antilles or nicaragua or niger or nigeria or oman or muscat or pakistan or panama or papua new guinea or new guinea or paraguay or peru or philippines or philipines or phillipines or phillippines or poland or "polish people's republic" or portugal or portuguese republic or puerto rico or romania or russia or russian federation or ussr or soviet union or union of soviet socialist republics or rwanda or ruanda or samoa or pacific islands or polynesia or samoan islands or navigator island or navigator islands or "sao tome and principe" or saudi arabia or senegal or serbia or seychelles or sierra leone or slovakia or slovak republic or slovenia or melanesia or solomon island or solomon islands or norfolk island or norfolk islands or somalia or south africa or south sudan or sri lanka or ceylon or "saint kitts and nevis" or "st. kitts and nevis" or saint lucia or "st. lucia" or "saint vincent and the grenadines" or saint vincent or "st. vincent" or grenadines or sudan or suriname or surinam or dutch guiana or netherlands guiana or syria or syrian arab republic or tajikistan or tadjikistan or tadzhikistan or tadzhik or tanzania or tanganyika or thailand or siam or timor leste or east timor or togo or togolese republic or tonga or "trinidad and tobago" or trinidad or tobago or tunisia or turkey or "turkey (republic)" or turkmenistan or turkmen or uganda or ukraine or uruguay or uzbekistan or uzbek or vanuatu or new hebrides or venezuela or vietnam or viet nam or middle east or west bank or gaza or palestine or yemen or yugoslavia or zambia or zimbabwe or northern rhodesia or global south or africa south of the sahara or sub-saharan africa or subsaharan africa or africa, central or central africa or africa, northern or north africa or northern africa or magreb or maghrib or sahara or africa, southern or southern africa or africa, eastern or east africa or eastern africa or africa, western or west africa or western africa or west indies or indian ocean islands or caribbean or central america or latin america or "south and central america" or south america or asia, central or central asia or asia, northern or north asia or northern asia or asia, southeastern or southeastern asia or south eastern asia or southeast asia or south east asia or asia, western or western asia or europe, eastern or east europe or eastern europe or developing country or developing countries or developing nation? or developing population? or developing world or less developed countr* or less developed nation? or less

developed population? or less developed world or lesser developed countr* or lesser developed nation? or lesser developed population? or lesser developed world or under developed countr* or under developed nation? or under developed population? or under developed world or underdeveloped countr* or underdeveloped nation? or underdeveloped population? or underdeveloped world or middle income countr* or middle income nation? or middle income population? or low income countr* or low income nation? or low income population? or lower income countr* or lower income nation? or lower income population? or underserved countr* or underserved nation? or underserved population? or underserved world or under served countr* or under served nation? or under served population? or under served world or deprived countr* or deprived nation? or deprived population? or deprived world or poor countr* or poor nation? or poor population? or poor world or poorer countr* or poorer nation? or poorer population? or poorer world or developing economy* or less developed economy* or lesser developed economy* or under developed economy* or underdeveloped economy* or middle income economy* or low income economy* or lower income economy* or low gdp or low gnp or low gross domestic or low gross national or lower gdp or lower gnp or lower gross domestic or lower gross national or lmic or lmics or third world or lami countr* or transitional countr* or emerging economies or emerging nation?).ti,ab,sh,kf. (1919265)

17 (afghan or afghans or afghani or albanian? algerian? or american samoan? or angolan? or antiguan? or barbudan? or argentine? or argentinian? or argentinean? or armenian? or aruban? or azerbaijani? or bahraini? or bangladeshi? or bangalees or bajan? or belarusian? or byelorussian? or belizean? or beninese? or bhutanese or bolivian? or bosnian? or botswana or batswana or brazilian? or brasilian? or bulgarian? or burkinabe or burkinese or burundian? or cape verdean? or cabo verdean? or cambodian? or khmer or cameroonian? or central african? or chadian? or chilean? or chinese or colombian? or comorian? or congolese or costa rican? or ivorian? or croatian? or cuban? or cypriot? or czech? or djiboutian? or dominican? or ecuadorian? or egyptian? or salvadoran? or equatorial guinean? or equatoguinean? or eritrean? or estonian? or swazi? or swati? or ethiopian? or fijian or gabonese or gabonaise or gambian? or georgian? or ghanaian? or gibraltarian? or greek? or grenadian? or guamanian? or guatemalan? or guinean? or bissau guinean? or guyanese or haitian? or honduran? or hungarian? or indian? or indonesian? or iranian? or iraqian? or iraqi? or manx or jamaican? or jordanian? or kazakhstani? or kenyan? or kirabati or kirabatian? or north korean? or korean? or kosovar? or kosovan? or kyrgyz* or lao or laotian? or latvian? or lebanese or lesothan? or lesothonian? or mosotho or basotho or liberian? or libyan? or lithuanian? or macanese or macedonian? or malagasy or madagascan? or malawian? or malaysian? or maldivian? or malian? or maltese or marshallese? or mauritanian? or mauritian? or mexican? or micronesian? or moldovan? or mongolian? or mongol or montenegrin? or moroccan? or mozambican? or burmese or myanma or namibian? or nauruan? or nepali or nepalese or netherlands antillean? or nicaraguan? or nigerien? or nigerian? or northern mariana islander? or mariana? or omani? or pakistani? or palauan? or panamanian? or papua new guinean? or paraguayan? or peruvian? or philippine? or philipine? or phillipine? or phillippine? or filipino? or filipina? or polish or pole or poles or portuguese or puerto rican? or romanian? or russian? or soviet people or soviet population or rwandan? or rwandese or ruandan? or ruandese or samoan? or sao tomean? or santomean? or saudi arabian? or saudi? or senegalese or serbian? or montenegrin? or seychellois or seychelloise? or sierra leonean? or slovak? or slovene? or solomon islander? or somali? or south african? or south sudanese or sri lankan? or ceylonese or kittitian? or nevisian? or saint lucian? or vincentian? or sudanese or surinamese? or syrian? or tajik? or tajikistani? or tanzanian? or tanganyikan? or thai or timorese? or togolese or tongan? or trinidadian? or tobagonian? or tunisian? or turk? or turkish or turkmen? or tuvaluan? or ugandan? or ukrainian? or uruguayan? or uzbek? or vanuatu* or venezuelan? or vietnamese or yemeni? or yemenite? or yemenese or yugoslav? or yugoslavian? or zambian? or zimbabwean?).ti,ab,sh,kf. (840691)

18 16 or 17 (2283252)

19 randomized controlled trial.pt. (509754)

20 controlled clinical trial.pt. (93762)

21 randomized.ab. (486234)

22 randomised.ab. (97082)

23 placebo.ab. (209447)

24 clinical trials as topic.sh. (192103)

25 randomly.ab. (337079)

26 trial.ti. (221842)

27 19 or 20 or 21 or 22 or 23 or 24 or 25 or 26 (1337740)

28 exp animals/ not humans.sh. (4718707)

29 27 not 28 (1233028)

30 15 and 18 and 29 (2569)

31 exp Child/ (1906042)

32 exp Adult/ (7189354)

33 31 not (31 and 32) (1210187)

34 30 not 33 (2343)

35 limit 34 to yr="1990 -Current" (2335)

****

2.2. PRISMA flow diagram

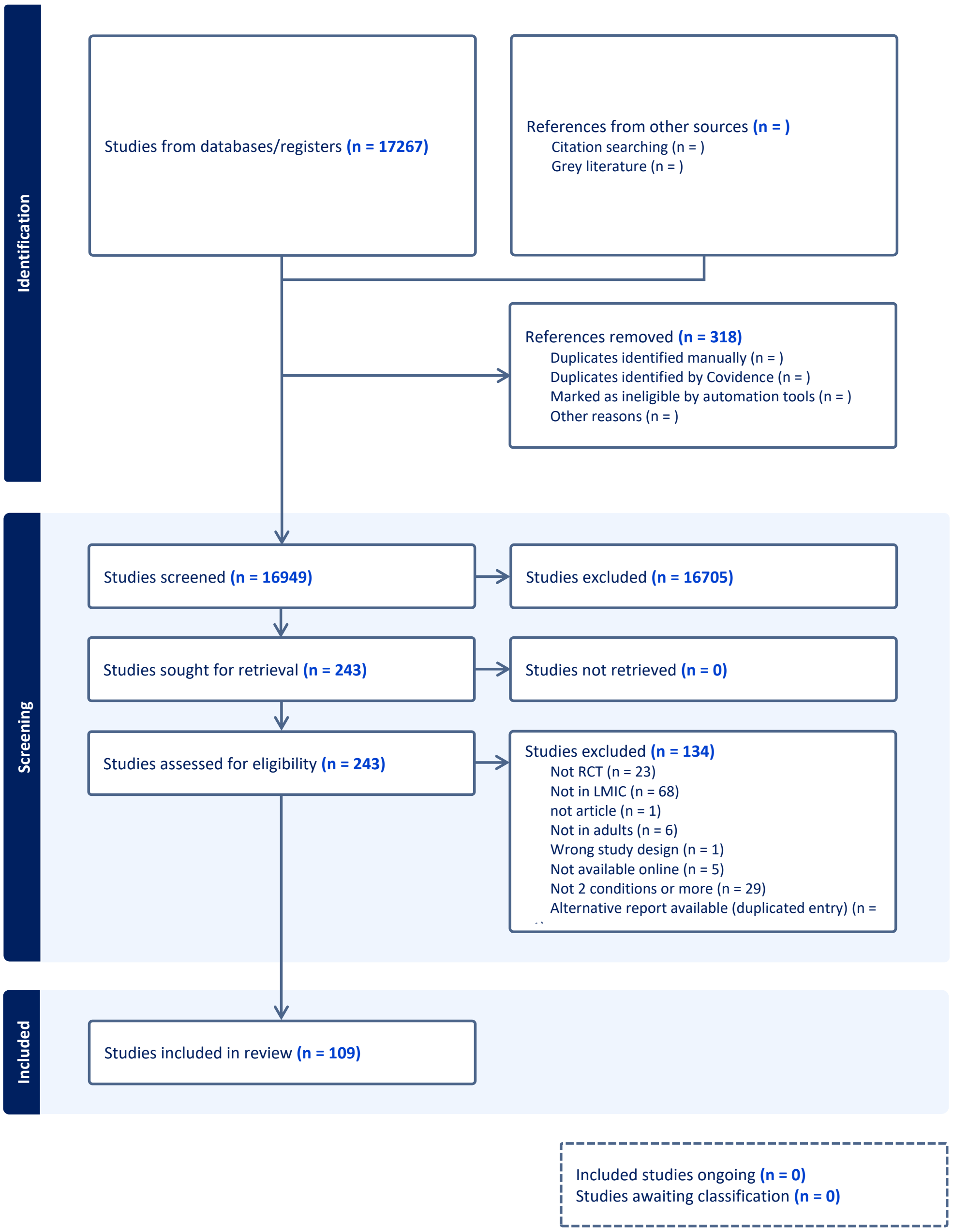

2.3. Table of characteristics of included studies

Study ID	Title	Country	Target conditions
Wang 2014	Effect of amlodipine plus atorvastatin calcium on hypertension patients complicated with hyperlipidaemia	China	Hypertension and hyperlipidaemia
Sanjeev 2013	Nevirapine vs. efavirenz-based ART regimens in antiretroviral-naive patients with HIV and tuberculosis infections in India: a pilot study	India	HIV and TB
Liang 2013	Effect of laparoscopic Roux-en-Y gastric bypass surgery on type 2 DM with hypertension: a randomized controlled trial	China	T2DM, obesity, hypertension
Li 2015	Observation of efficacy of trimetazidine in treatment of CHD combined with diabetes	China	Coronary heart disease and diabetes
Wang 2020	A Chinese herbal formula shows beneficial effects on comorbid depression and coronary heart disease based on the philosophy of psycho-cardiology	China	Depression and Coronary Heart disease
Xinju 2017	The efficacy of internet-based cognitive behaviour therapy on blood pressure for comorbid hypertension and insomnia	China	Hypertension and insomnia
Xin 2002	A Clinical Trial of Paroxetine and Psychotherapy in Patients with Poststroke Depression and Anxiety	China	Acute brain stroke, depression, anxiety
Freitas Júnior 2010	Estudo comparativo dos efeitos agudos do sildenafil e nitroprussiato de sódio sobre a hipertensão pulmonar de pacientes com insuficiência cardíaca avançada	Brazil	Pulmonary hypertension and heart failure
Duarte 2011	Síndrome da apnéia do sono na acromegalia: impacto do tratamento sobre o metabolismo dos carboidratos	Brazil	Acromegaly and sleep apnoea
Mokhber 2011	[Comparison of desipramine and sertraline in the treatment of depression in patients suffering from Alzheimer disease]	Iran	Depression in Alzheimer's patients

Morad Rasouli 2009	[Effects of cognitive behavioural group therapy in male opioid dependent patients comorbid with major depressive disorder]	Iran	Depression in opioid-dependent men receiving methadone treatment
Huiying 2012	Influence of comprehensive nursing intervention on pulmonary function and depressive mood of patients with COPD and depression	China	COPD and depression
Bündchen 2010	Ausência de influência da massa corporal na redução da pressão arterial após exercício físico	Brazil	Hypertension and Overweight or Obesity
An-Lin 2006	Effects of antidepressant therapy in patients with suspected angina pectoris and negative coronary angiogram complicating comorbid depression	China	Suspected angina pectoris with negative coronary angiogram and depression
Aiyun 2017	A study on the treatment of depression and anxiety in elderly patients by Shu gan jie yu capsule combined with Ascitalopram	China	Anxiety, Depression
Buranakitjaroen 2007	Efficacy and safety of sildenafil in Asian males with erectile dysfunction and cardiovascular risk	Thailand, Malaysia	Erectile dysfunction, with one or more of HT, dyslipidaemia, T2DM
Vanelle 2006	A double-blind RCT of amisulpride versus olanzapine for 2 months in the treatment of subjects with schizophrenia and comorbid depression	France, Italy, and Tunisia.	Schizophrenia and Comorbid depression
Dong 2005	Anxiolytic intervention for coronary heart disease patients combined with anxiety disorder. [Chinese]	China	coronary heart disease and anxiety
Manosuthi 2005	Efavirenz levels and 24-week efficacy in HIV-infected patients with tuberculosis receiving highly active ART and rifampicin	Thailand	HIV and TB
Liu 2005	Effect and safety of the treatment for comorbid anxiety and depression by use of short-term and low-dose olanzapine in combination with fluoxetine.	China	Anxiety and depression
Emre 2004	Rivastigmine for dementia associated with Parkinson's disease	12 countries	Dementia and Parkinsons
Miti 2003	Integration of TB treatment in a community-based home care programme for persons living with HIV/AIDS in Ndola, Zambia	Zambia	HIV and TB

Li 2009	Beneficial impact of Xuezhikang on cardiovascular events and mortality in elderly hypertensive patients with previous myocardial infarction from the China Coronary Secondary Prevention Study (CCSPS)	China (19 provinces)	HT and Myocardial inaction
NCT 2013	Chinese Older Adults-Collaboration in Health (COACH)Study	China	Hypertension and depression
Poirier 2009	Adding prompt revascularization to medical therapy did not reduce mortality or CV events in patients with type 2 diabetes and CAD	6 countries	Type 2 diabetes and heart disease
Moosa 2012	Antidepressants versus interpersonal psychotherapy in treating depression in HIV-positive patients	South Africa	Depression in HIV patients receiving antiretroviral treatment
PrayGod 2012	The effect of energy-protein supplementation on weight, body composition and handgrip strength among pulmonary tuberculosis HIV-co-infected patients: randomised controlled trial in Mwanza, Tanzania	Tanzania	TB and HIV
Greenfield 2010	Integrated Management of Physician-delivered Alcohol Care for Tuberculosis Patients: Design and Implementation	Russia	Alcohol Use in people with TB
Nyamathi 2012	Impact of an ASHA intervention on depressive symptoms among rural women living with AIDS in India	India	Depression in women living with AIDS
Durovni 2010	The implementation of isoniazid preventive therapy in HIV clinics: the experience from the TB/HIV in Rio (THRio) study	Brazil	TB in HIV patients
Shah 2012	Improving diabetes care: Multi-component cardiovascular disease risk reduction strategies for people with diabetes in South Asia: CARRS	India and Pakistan	Risk of CVD in patients with Diabetes
Heeley 2011	The Sleep Apnoea cardioVascular Endpoints (SAVE) study: 1000 recruited patients	5 countries	OSA, cardiovascular disease
Peltzer 2013	Screening and brief interventions for hazardous and harmful alcohol use among patients with active tuberculosis attending primary public care clinics in South Africa: results from a cluster randomized controlled trial	South Africa	Alcohol misuse among active TB patients

Palavras 2015	Comparing cognitive behavioural therapy for eating disorders integrated with behavioural weight loss therapy to cognitive behavioural therapy-enhanced alone in overweight or obese people with bulimia nervosa or binge eating	Brazil	Bulimia nervosa/eating disorders and overweight/obesity
NCT 2016a	Diabetes Complication Control in Community Clinics (D4C) Trial	China	CVD risk in patients with diabetes
Fairall 2016	Educational Outreach with an Integrated Clinical Tool for Nurse-Led Non-communicable Chronic Disease Management in Primary Care in South Africa: A Pragmatic Cluster Randomised Controlled Trial	South Africa	Hypertension, Diabetes, Chronic respiratory disease, Depression
NCT 2015	The Effect of N- Acetylcysteine on Inflammatory and Oxidative Stress Biomarkers	Brazil	Bipolar Disorder
NCT 2014a	Effect of Supplementary Vitamin D in Patients With Diabetes Mellitus and Pulmonary Tuberculosis	Pakistan	Diabetes and Pulmonary TB
NCT 2016b	Effectiveness of a Mobile Technology Intervention for the Treatment of Depression	Brazil	Depressive symptoms and co-morbid diabetes and/or hypertension
Asl 2014	Effectiveness of Mindfulness-Based Cognitive Therapy for Co-Morbid Depression in Drug-Dependent Males	Iran	Depression in drug dependent population
NCT 2016c	Effectiveness of Nurse-delivered Care for Adherence/Mood in HIV in South Africa	South Africa	HIV and Depressive Symptoms
Da Silva 2015	Effects of exercise training and photobiomodulation therapy (EXTRAPHOTO) on pain in women with fibromyalgia and temporomandibular disorder: protocol for RCT	Brazil	Women with fibromyalgia and temporomandibular disorder
Pervane Vural 2016	Effects of Mirror Therapy in Stroke Patients With Complex Regional Pain Syndrome Type 1: A Randomized Controlled Study	Turkey	First-time stroke and simultaneous complex regional pain syndrome
NCT 2014b	Improving Mental Health Through Integration With Primary Care in Rural Karnataka	India	Depression or anxiety and co-morbid diabetes, or cardiovascular disease

Hasanpour-Dehkordi 2016	Effects of yoga on physiological indices, anxiety and social functioning in multiple sclerosis patients: a randomized trial	Iran	Multiple sclerosis (MS), Anxiety
Huo 2015	Efficacy of folic acid therapy in primary prevention of stroke among adults with hypertension in China: The CSPPT randomized clinical trial	China	Stroke (prevention of stroke), Hypertension
Safi 2015	A Fresh Look at the Mechanisms of Progressive Muscle Relaxation on Depression in Female Patients with Multiple Sclerosis	Iran	Depression, Multiple sclerosis (MS)
Petersen 2014	A group-based counselling intervention for depression comorbid with HIV/AIDS using a task shifting approach in South Africa	South Africa	Depression and HIV/AIDS
Basheti 2016	Home medication management review in outpatients with chronic diseases in Jordan: a randomized control trial	Jordan	Mean (SD) number of conditions per patient was 3.97 (1.56)
Song 2015	Influence of different exercise nursing intervention plans on blood glucose level of type 2 DM patients complicated with obesity	China	Type 2 diabetes mellitus (T2DM) and obesity
NCT 2016d	Integrated Primary Care for Diabetes and Cardiovascular Disease	Brazil	Cardiovascular disease and diabetes
Wagner 2014	INtegration of DEPression Treatment into HIV Care in Uganda (INDEPTH-Uganda): study protocol for a randomized controlled trial	Uganda	HIV and depression
Hu 2015	Suan zao ren tang in combination with zhi zi chi tang as a treatment protocol for insomniacs with anxiety: parallel-controlled RCT	China	Insomnia and anxiety
Bolton 2014	A transdiagnostic community-based mental health treatment for comorbid disorders: development and outcomes of a randomized controlled trial among Burmese refugees in Thailand	Thailand	Mental Health and Alcohol Use
Budnevsky 2016	[Pulmonary rehabilitation as an effective method for optimizing therapeutic and preventive measures in patients with chronic obstructive pulmonary disease concurrent with metabolic syndrome]	Russia	Chronic obstructive pulmonary disease (COPD) concurrent with metabolic syndrome (MS)
Liu 2015	Pulsed electromagnetic fields for postmenopausal osteoporosis and concomitant lumbar osteoarthritis in southwest China using proximal femur	China	Postmenopausal osteoporosis, lumbar osteoarthritis

	bone mineral density as the primary endpoint: Study protocol for a randomized controlled trial		
Gurevich 2015	[Psychotherapy in patients with alcohol dependence and comorbid endogenous pathology]	Russia	Alcohol dependence, developed against paranoid schizophrenia
Sevostyanova 2018	[Combined rehabilitation in the patients presenting with dorsopathies of the lumbar spine and concomitant ibs based at a therapeutic clinic]	Russia	Dorsopathy of the lumbar spine, combined with IBS
Majeed 2018	Bacillus coagulans MTCC 5856 for the management of major depression with irritable bowel syndrome: A randomised, double-blind, placebo controlled, multi-centre, pilot clinical study	India	Irritable bowel syndrome (IBS), Major depressive disorder (MDD)
Thapinta 2017	Cognitive Behaviour Therapy Self-Help Booklet to Decrease Depression and Alcohol Use among People with Alcohol Dependence in Thailand	Thailand	Depression, Alcohol dependence
NCT 2018a	Cognitive Processing Therapy Versus Medication for the Treatment of Post Traumatic Stress Disorder & Substance Use Disorder Egyptian Patients	Egypt	Substance Use Disorder, Post-Traumatic Stress Disorder
Fairall 2018	Collaborative care for the detection and management of depression among adults receiving antiretroviral therapy in South Africa: study protocol for the CobALT randomised controlled trial	South Africa	depression among HIV patients receiving antiretroviral therapy
Petersen 2018	Collaborative care for the detection and management of depression among adults with hypertension in South Africa: protocol for the PRIME-SA RCT	South Africa	Depression and hypertension
Myers 2018	Comparing dedicated and designated models of integrating mental health into chronic disease care: study protocol for a cluster RCT	South Africa	Alcohol misuse or depression in diabetic or HIV patients
NCT 2017a	Effect of Different Exercises on Musculoskeletal Pain, Glucose Level and Quality of Life Among Patients With Diabetes	South Africa	Diabetes, Depression
NCT 2018b	Effect of Dulaglutide on Liver Fat in Patients With Type 2 Diabetes and Nonalcoholic Fatty Liver Disease	India	Non-Alcoholic Fatty Liver Disease, Type 2 Diabetes Mellitus

Zhang 2017	Effect of HBM Rehabilitation Exercises on Depression, Anxiety and Health Belief in Elderly Patients with Osteoporotic Fracture	China	Depression, anxiety in elderly with osteoporotic fracture
Tu 2018	Hypertension management for community-dwelling older people with diabetes in Nanchang, China: study protocol for a cluster randomized controlled trial	China	Hypertension in older people with type 2 diabetes
Abas 2018	Feasibility and acceptability of a task-shifted intervention to enhance adherence to HIV medication and improve depression in people living with HIV in Zimbabwe, a low-income country in sub-Saharan Africa	Zimbabwe	HIV and depression
Valiani 2018	The effect of auriculotherapy on stress, anxiety, and depression in MS patients: A double blind randomized clinical control trial (parallel design)	Iran	Multiple sclerosis (MS), Anxiety, Depression
NCT 2017b	Effectiveness of a mHealth Intervention for the Treatment of Depression in People with Diabetes or Hypertension in Peru	Peru	Depression, Diabetes, Hypertension
Ahmadi 2018	The effectiveness of different singly administered high doses of buprenorphine in reducing suicidal ideation in acutely depressed people with co-morbid opiate dependence: double-blind RCT	Iran	People living with severe Opioid Use Disorder and depression
Bahrani 2017	The effectiveness of mindfulness-integrated cbt on depression, anxiety, and stress in females with multiple sclerosis: A single blind RCT	Iran	Multiple Sclerosis with Depression, Anxiety, and 'Stress'
Rashidi 2018	The Effects of Acute Multisession Bifrontal Transcranial Direct Current Stimulation on Intractable Tinnitus and comorbid depression and anxiety: A Study Protocol for Randomized Controlled Trial	Iran	Intractable Tinnitus and comorbid depression and anxiety
Djazayery 2018	The effects of alcoholic extract of saffron (Crocus satious L.) on mild to moderate comorbid depression-anxiety, sleep quality, and life satisfaction in type 2 diabetes mellitus: A double-blind, placebo-controlled RCT	Iran	Type 2 diabetic patients suffering from mild to moderate anxiety and depression
Moazen-Zadeh 2018	Effects of Saffron on Cognition, Anxiety, and Depression in Patients Undergoing Coronary Artery Bypass Grafting: A Double-Blind Placebo-RCT	Iran	Depression and anxiety in patients undergoing CABG

Sudfeld 2017	Efficacy of vitamin D3 supplementation in reducing incidence of pulmonary tuberculosis and mortality among HIV-infected Tanzanian adults initiating antiretroviral therapy: study protocol for a randomized controlled trial	Tanzania	pulmonary tuberculosis among HIV-infected
Noroozi 2017	Improving Depression, and Quality of Life in Patients with Type 2 Diabetes: Using Group Cognitive Behavior Therapy	Iran	Type 2 diabetes, Depression
Bongomin 2018	Integrated versus referred management of cardiovascular disease (CVD) risk factors for HIV-positive patients on antiretroviral therapy in Swaziland	Swaziland (Eswatini)	HIV, Cardiovascular disease risk factors
Keshavarz 2018	Omega-3 supplementation effects on body weight and depression among dieter women with co-morbidity of depression and obesity compared with the placebo: A randomized clinical trial	Iran	depression and obesity
Kowalski 2017	The INtegrating DEPrEssioN and Diabetes treatmENT (INDEPENDENT) study: Design and methods to address mental healthcare gaps in India	India	Depression and Diabetes
ISRCTN 2017	An Intervention Program to Reduce the Risk Factors of Metabolic Syndrome in Malaysian Female University Staff with Polycystic Ovarian Syndrome	Malaysia	Polycystic Ovarian Syndrome (PCOS), Metabolic Syndrome
Steinert 2017	Resource activation for treating post-traumatic stress disorder, co-morbid symptoms and impaired functioning: a RCT in Cambodia	Cambodia	Post-traumatic stress disorder (PTSD), Anxiety, Depression
Zemestani 2017	A Preliminary Investigation on the Effectiveness of Unified and Transdiagnostic CBT for Patients with Comorbid Depression and Anxiety	Iran	Depression and anxiety
Jha 2017 and Prabhakaran 2018	Protocol for the mWellcare trial: a multicentre, cluster RCT to compare the effectiveness of mWellcare, an mHealth system for an integrated management of patients with hypertension and diabetes, versus EUC	India	Hypertension and diabetes
Chen 2018	Protocol of an ongoing randomized controlled trial of care management for comorbid depression and hypertension: the Chinese Older Adult Collaborations in Health (COACH) study	China	depression and hypertension

Miller 2018	A scalable, integrated intervention to engage people who inject drugs in HIV care and medication-assisted treatment (HPTN 074): a RCT phase 3 feasibility and efficacy study	Ukraine; Vietnam; Indonesia	People who inject drugs: HIV-infected and their HIV-uninfected injection partners.
ReBEC 2018	Vitamin D action in the treatment of Depression and reduction of Cardiovascular Risk	Brazil	Depression, Cardiovascular risk
Caetano 2018	Trial-Based Cognitive Therapy: Efficacy of a New CBT Approach for Treating Social Anxiety Disorder with Comorbid Depression	Brazil	Generalized social anxiety disorder in comorbid depression
ISRCTN 2019a	A collaborative care psychosocial intervention for lowering blood pressure among old adults with hypertension and depression in primary care in Guarulhos, Brazil: the PROACTIVE-H study	Brazil	Hypertension and depression in older adults.
Kane 2020	Common Elements Treatment Approach (CETA) for unhealthy alcohol use among persons with HIV in Zambia: Study protocol	Zambia	HIV and high-risk alcohol use and possible mental health
Furtado 2019	Dapagliflozin and Cardiovascular Outcomes in Patients with Type 2 Diabetes Mellitus and Previous Myocardial Infarction	33 countries	Type II DM and those with previous MI
Khan 2019	Efficacy of eye movement desensitization & reprocessing versus cognitive behavioural therapy in PTSD Symptoms: Study protocol for RCT	Pakistan	PTSD and depressive symptoms
Pirnia 2019	Evaluation of the effectiveness of auricular acupuncture in suicidal ideation and cortisol level in dysthymic patients with comorbid opiate use	Iran	Dysthymic patients with comorbid opiate use disorders
ISRCTN 2019b	Determination of the Effect of Crude Oil on Erectile Dysfunction in Men with Multiple Sclerosis	Iran	Men with multiple sclerosis and erectile dysfunction
Li 2019	Effect of a patient education and rehabilitation program on anxiety, depression and quality of life in muscle invasive bladder cancer patients	China	Anxiety, depression, and bladder cancer
Abdollahi 2020	The effect of aromatherapy with bitter orange (Citrus aurantium) extract on anxiety and fatigue in type 2 diabetic patients	Iran	anxiety and fatigue in people with type 2 diabetes

Levy 2019	Implementation research for public sector mental health care scale-up (SMART-DAPPER): a sequential multiple, assignment randomized trial	Kenya	Major depressive disorder and PTSD
Negaresh 2019	Effect of Short-Term Interval Exercise Training on Fatigue, Depression, and Fitness in Normal Weight vs. Overweight Person with Multiple Sclerosis	Iran	Overweight and Multiple sclerosis
Zemestani 2020	Effectiveness of mindfulness-based cognitive therapy for comorbid depression and anxiety in pregnancy: a randomized controlled trial	Iran	Comorbid depression and anxiety (in pregnant women)
Cruz 2019	Morbidity in patients with uncontrolled hypertension and obstructive sleep apnoea: baseline profile of the MORPHEOS study	Brazil	Uncontrolled hypertension and Significant sleep apnoea
Mutyambizi-Mafunda 2019	Integrating a brief mental health intervention into primary care services for patients with HIV and diabetes in South Africa: study protocol	South Africa	Diabetes, HIV, Depression, Hazardous alcohol use
Becerril-Alarcon 2019	Inulin Supplementation Reduces Systolic Blood Pressure in Women with Breast Cancer Undergoing Neoadjuvant Chemotherapy	Mexico	Blood pressure in women with breast cancer receiving chemotherapy
Joska 2020	Nurse-Delivered Cognitive Behavioral Therapy for Adherence and Depression Among People Living With HIV (the Ziphamandla Study): Protocol for a Randomized Controlled Trial	South Africa	HIV and depression
Citrome 2019	Olanzapine Plus Samidorphan in Subjects with Schizophrenia and Comorbid Alcohol Use Disorder: Rationale and Design for a Phase II rct	US, Bulgaria, Poland	Schizophrenia, Alcohol Use Disorder
Akhondzadeh 2020	A placebo controlled RCT of Crocus sativus L. (saffron) on depression and food craving among overweight women with mild to moderate depression	Iran	depression and obesity (in women)
Tu 2020	A transitional care intervention for hypertension control for older people with diabetes: A cluster randomized controlled trial	China	Hypertension control in older people with diabetes
NCT 2019	Vortioxetine vs Sertraline in Mexicans	Mexico	Type II diabetes and Depression

Appendix 3: Ethics committee approvals

Organisation	Country	Approving committee/ department
University of York, York	United Kingdom	Health Sciences Research Governance Committee (HSRGC/2020/409/D: COSMOS)
Afghan Thoughts for Development and Change, Kabul	Afghanistan	Department of Research and Development
Bangladesh University of Health Sciences, Dhaka	Bangladesh	Department of Public Health Sciences, Centre for Injury Prevention and Research Bangladesh
Association Mahidol de Sante Publique, Ouagadougou	Burkina Faso	Comite D'éthique Pour La Recherche En Sante
Community and Family Aid Foundation Ghana, Newtown-Accra	Ghana	CAFAF/20/COSMOS /3 (Local Reference: Project 101/20)
Universidad Autónoma de Querétaro, Santiago de Querétaro	Mexico	C.U., 1 de febrero de 2021 DIP/56-2021
National Medical College, Birgunj	Nepal	Institutional Review Committee (IRC), Ref. F-NMC/518/077-078
University of Benin Teaching Hospital, Benin City	Nigeria	Health Research Ethics Committee, ADM/E 22/A/VOL. VII/14830970
Rawalpindi Medical University & Allied Hospitals, Rawalpindi	Pakistan	Research and Ethical Committee, 247/IREF/RMU/2020
PRISMA ONG, Lima	Peru	Comité Institucional de Ética en Investigación, CE0981.20
Ministerie van Volksgezondheid in Suriname	Suriname	Direction Centrale Administratie

Appendix 4: Examples codes for outcomes added from qualitative interviews.

Country	Quoted passage	Extracted outcome
Afghanistan	In my country, there are also limited health care providers who can rightly diagnose and treat these conditions. These all challenges made me worried about my health conditions.	Healthcare access
Afghanistan	Follow the doctor’s advice and keep the patient tracking his medication and treatment plan.	Adherence to treatment
Bangladesh	a lot of things were restricted, like food.	Diet
Bangladesh	encourage him emotionally to live a happy and healthy life.	Psychological wellbeing
Burkina Faso	So, I am more afraid.	Health anxiety
Burkina Faso	The impact for us is the high cost of care.	Healthcare cost
Ghana	We want treatment to help him heal and resume his normal activities.	Functioning/ADL
Ghana	I could not sleep.	Sleep
Mexico	And now I have my three haemodialysis sessions, and the truth, the haemodialysis, the diet, and exercise, have lifted me up in a phenomenal way.	Perceived health
Mexico	I hope that, well, I have a better life, stability	Health-related quality of life
Nepal	Accessibility of check-up in every household for screening such disease.	Timely screening
Nepal	She does not want to spend on me. My son sends money from abroad to daughter-in-law, but she doesn’t want to spend those on my treatment.	Carer burden
Nigeria	I’ve not been taking it much, that I take you see at time I don’t take it every time.	Adherence to treatment
Nigeria	So he now changed my drugs and then I was now complaining that the thing became severe.	Perceived health
Pakistan	Now she gets angry.	Agitation
Pakistan	Good quality medicines should be provided and used before, during and after treatment for both rich and poor without any discrimination.	Healthcare quality
Peru	I would hope the best not, to be able to live normal, to be able to make normal life.	Health-related quality of life

Peru	the CT scan was one hundred [soles] and so many, and the other was almost 300 soles, so at that time I did not have…	Healthcare cost
Suriname	She does not want additional medication prescribed or that the doses be increased.	Reduced medication
Suriname	Physical therapist taught me was to accept my disabilities. It was hard because I realized that my life would never be the same.	Acceptance of illness

Appendix 5: Prevention and Treatment outcomes from outcome generation and Delphi R1 stages, classified using Dodd's taxonomy.

S no.	Outcome	Help text	Core areas*	Williamson/Clarke (revised scale) *
**Prevention outcomes**				
1	Adherence to treatment	The extent to which a person keeps to a recommended treatment, such as taking prescribed drugs or following a diet or exercise programme	Life impact	Delivery of Care
2	Adverse events	Any unexpected or unintended negative consequences of an intervention, for example, allergic reaction to a drug, or injury from a recommended exercise programme	Adverse events	Adverse Events/ Effects
3	Cognitive function	Thinking abilities including ability to concentrate, remember, and to have awareness of others and the surrounding environment	Life impact	Cognitive Functioning
4	Comorbidity	Having more than one long term illness or condition occurring at the same time. Preventing comorbidity refers to preventing development of a new illness alongside the original health problem	Physiological or clinical	General Outcomes
5	Cost effectiveness**	The value for money of a treatment	Resource use	Economic
6	Cardiovascular risk	A person's future risk of developing problems with the heart or blood circulation, such as a heart attack or stroke	Physiological or clinical	Cardiac Outcomes
7	Cardiovascular event	Problems with the function of the heart or blood vessels such as a heart attack, stroke, abnormal heart rhythm, chest pain caused by reduced blood flow to the heart muscle, or heart failure	Physiological or clinical	Cardiac Outcomes
8	Diet	A person's regular eating habits	Physiological or clinical	Metabolism and Nutrition Outcomes
9	Exercise tolerance	How much exercise an individual can manage	Life impact	Physical Functioning
10	Fatigue	Extreme tiredness or lack of energy	Life impact	Physical Functioning

11	Health literacy	The ability to gather, read, understand, and use healthcare information to make informed decisions about one's own health	Life impact	Personal Circumstances
12	Healthcare use**	How much a person uses a healthcare service including visiting a doctor or other health professional or going to hospital	Life impact	Delivery of Care
13	Health-related quality of life	The impact of physical or mental health conditions and their treatments on how a person views their quality of life or wellbeing	Life impact	Perceived Health Status
14	Prevention of hypertension	Prevention of developing high blood pressure	Physiological or clinical	Vascular Outcomes
15	Psychological wellbeing	A person's emotional and mental wellbeing, including problems with low mood or anxiety	Life impact	Emotional Functioning/Wellbeing
16	Death	-	Death	Mortality/Survival
17	Organ damage	Some form of injury or harm leading to an organ such as lungs or kidneys not working well	Physiological or clinical	General Outcomes
18	Pain	-	Physiological or clinical	General Outcomes
19	Quality of life	A person's view of their emotional, physical, material, and social well-being. It includes the degree to which a person feels they are healthy, comfortable, and able to participate in or enjoy life events.	Life impact	Global Quality of Life
20	Reduced medication	Reducing the dose or number of drugs a person requires	Life impact	Delivery of Care
21	Timely screening	An activity that detects a health problem (or risk of a health problem) early, so it can be prevented or treated.	Resource use	Need for Further Intervention
22	Treatment satisfaction	How content a patient is with the treatment they have received, and how well they think it was delivered	Life impact	Delivery of Care
23	Weight	-	Physiological or clinical	Metabolism and Nutrition Outcomes

24	Obesity	A condition in which a person's weight is above a certain level, which is associated with risks to health, including increased risk of heart disease and diabetes	Physiological or clinical	Metabolism and Nutrition Outcomes
**Additional prevention outcomes added at Delphi R1**				
1	Carer burden	Emotional, physical, and psychological stress or burden that caregivers experience when looking after a person with health problems. Caregivers may be family members, friends or health and social care workers	Resource use	Societal/Care Burden
2	Chronic disease self-management	The ability of an individual to undertake activities by themselves to manage their long-term health conditions	Life impact	Delivery of Care
3	Early detection	When a condition is identified early enough to prevent it from developing fully	Physiological or clinical	General Outcomes
4	Functioning/ADL	A person's ability to carry out activities of daily living for themselves e.g., washing or shopping	Life impact	Role Functioning; Physical Functioning; Social Functioning
5	Health anxiety	A fear or worry of becoming ill	Life impact	Perceived Health Status
6	Health risk behaviour	Behaviours that increase the risk of disease or injury e.g., smoking	Physiological or clinical	General Outcomes
7	Health seeking behaviour	Activities undertaken by an individual in order to receive treatment for a health problem	Life impact	Delivery of Care
8	Income	The money that a person receives on a regular basis in exchange for working or providing services	Life impact	Personal circumstances
9	Loneliness	A feeling of being alone caused by a lack of social contact	Life impact	Social Functioning
10	Perceived health	A person's own assessment of how good they feel their overall health is	Life impact	Perceived Health Status

11	Self-efficacy	An individual's belief to have the ability to do what is necessary to achieve a desired outcome (e.g., symptom control).	Life impact	Personal circumstances
12	Social functionality	How an individual fulfils their roles in and engages with their social environment (e.g., family, work)	Life impact	Social Functioning
**Treatment outcomes**				
1	Acceptance of illness	Being able to come to terms with a diagnosis	Life impact	Emotional Functioning/Wellbeing
2	Adherence to treatment	The extent to which a person keeps to a recommended treatment, such as taking prescribed drugs or following a diet or exercise programme	Life impact	Delivery of Care
3	Adverse events	Any unexpected or unintended negative consequences of an intervention, for example, allergic reaction to a drug, or injury from a recommended exercise programme	Adverse events	Adverse Events/Effects
4	Aggression	A hostile behaviour towards another person that can lead to emotional or physical harm	Life impact	Emotional Functioning/Wellbeing
5	Agitation	Level of restlessness	Life impact	Emotional Functioning/Wellbeing
6	Appetite	-	Physiological or clinical	Metabolism and Nutrition Outcomes
7	Balance	A person's ability to stand or move without swaying or falling over	Physiological or clinical	Nervous System Outcomes
8	Carer burden***	Emotional, physical, and psychological stress or burden that caregivers experience when looking after a person with health problems. Caregivers may be family members, friends or health and social care workers	Resource use	Societal/Care Burden
9	Cognitive function	Thinking abilities including ability to concentrate, remember, and to have awareness of others and the surrounding environment	Life impact	Cognitive Functioning

10	Comorbidity	Having more than one long term illness or condition occurring at the same time	Physiological or clinical	General Outcomes
11	Cost effectiveness	The value for money of a treatment	Resource use	Economic
12	Cardiac event risk	A person's future risk of developing problems with the heart or blood circulation, such as a heart attack or stroke	Physiological or clinical	Cardiac Outcomes
13	Cardiovascular event	Problems with the function of the heart or blood vessels such as a heart attack, stroke, abnormal heart rhythm, chest pain caused by reduced blood flow to the heart muscles, or heart failure	Physiological or clinical	Cardiac Outcomes
14	Diet	A person's regular eating habits	Physiological or clinical	Metabolism and Nutrition Outcomes
15	Domestic violence	Forms of abuse including physical and emotional, which occur within couple relationships or between family members in the home setting	Life impact	Personal circumstances
16	Emotional regulation	The ability to adjust one's emotions in response to a situation	Life impact	Emotional Functioning/Wellbeing
17	Falls risk	The risk of falling, based on assessing multiple risk factors, such as problems with thinking and memory, vision, balance, or mobility. Falls tend to occur more in older people.	Life impact	Physical Functioning
18	Fatigue	Extreme tiredness or lack of energy	Life impact	Physical Functioning
19	Functioning/ADL	A person's ability to carry out activities of daily living for themselves e.g., washing or shopping	Life impact	Role Functioning; Physical Functioning; Social Functioning
20	Health anxiety	A fear or worry of becoming ill	Life impact	Perceived Health Status
21	Health literacy	The ability to gather, read, understand, and use healthcare information to make informed decisions about one's own health	Life impact	Personal Circumstances
22	Health risk behaviour	Behaviours that increase the risk of disease or injury e.g., smoking	Physiological or clinical	General Outcomes

23	Healthcare cost	The cost of a healthcare service that is paid either by the government, the patient, or their family	Resource use	Economic
24	Healthcare access	Being able to access health services to improve one's health and wellbeing	Life impact	Delivery of Care
25	Healthcare quality	The standard of healthcare provided by a service	Life impact	Delivery of Care
26	Healthcare staff communication	Written and spoken communication by healthcare staff to patients and caregivers about the patient's health and healthcare	Life impact	Delivery of Care
27	Healthcare use	How much a person uses a healthcare service including visiting a doctor or other health professional or going to hospital	Life impact	Delivery of Care
28	Hospital admission	When an individual is admitted onto a hospital ward as an inpatient	Resource use	Hospital
29	Health-related quality of life	The impact of physical or mental health conditions on how a person views their quality of life or wellbeing	Life impact	Perceived Health Status
30	Hypertension	High blood pressure	Physiological or clinical	Vascular Outcomes
31	Illness resolution	When a health problem has fully resolved leading to that aspect of health returning to normal	Physiological or clinical	General Outcomes
32	Illness stigma	The negative attitudes people have towards someone with a particular physical or mental health condition	Life impact	Emotional Functioning/Wellbeing
33	Illness under control	A health problem that is no longer getting worse due to a treatment(s)	Physiological or clinical	General Outcomes
34	Income	The money that a person receives on a regular basis in exchange for working or providing services	Life impact	Personal circumstances
35	Increase in symptoms	The increase in physical or mental features of a health problem such as pain or shortness of breath	Physiological or clinical	General Outcomes
36	Loneliness	A feeling of being alone caused by a lack of social contact	Life impact	Social Functioning

37	Psychological wellbeing	A person's emotional and mental wellbeing, including problems with low mood or anxiety	Life impact	Emotional Functioning/Wellbeing
38	Death	-	Death	Mortality/Survival
39	Nausea	Feeling sick	Physiological or clinical	Gastrointestinal Outcomes
40	Pain	-	Physiological or clinical	General Outcomes
41	Perceived health	A person's own assessment of how good they feel their overall health is	Life impact	Perceived Health Status
42	Physical activity	Any body movement including sitting, standing, walking, running	Physiological or clinical	General Outcomes
43	Quality of life	A person's view of their emotional, physical, material, and social well-being. It includes the degree to which a person feels they are healthy, comfortable, and able to participate in or enjoy life events.	Life impact	Global Quality of Life
44	Reduced medication	Reducing the dose or number of drugs a person requires	Life impact	Delivery of Care
45	Self-management	The tasks that individuals must undertake themselves to manage long term health conditions	Life impact	Role Functioning
46	Sleep quality	How well someone sleeps	Physiological or clinical	General Outcomes
47	Treatment satisfaction	How content a patient is with the treatment they have received, and how well they think it was delivered	Life impact	Delivery of Care
48	Weight	-	Physiological or clinical	Metabolism and Nutrition Outcomes
49	Obesity	A condition in which a person's weight is above a certain level, which is associated with risks to health, including increased risk of heart disease and diabetes	Physiological or clinical	Metabolism and Nutrition Outcomes

Additional treatment outcomes added at Delphi R1				
1	Continuity of care	-	Life impact	Delivery of Care
2	Frailty	-	Life impact	Social Functioning
3	Polypharmacy	The use of five or more routine medications - this can be over-the -counter, prescription, or complementary medicines	Physiological or clinical	General Outcomes
4	Self-esteem	An individual's own evaluation of their self-worth i.e., the confidence they have in themselves	Life impact	Emotional Functioning/Wellbeing
5	Social functionality	How an individual fulfils their roles in and engages with their social environment (e.g., family, work)	Life impact	Social Functioning
6	Treatment burden***	The impact of the demands of treatment regimens on patient wellbeing, functioning, and time	Life impact	Personal circumstances

* Dodd S, Clarke M, Becker L, Mavergames C, Fish R, Williamson PR. A taxonomy has been developed for outcomes in medical research to help improve knowledge discovery. Journal of clinical epidemiology. 2018;96:84-92.

** Under the prevention outcomes, there were discussions around the inclusion of 'Cost effectiveness' and 'Healthcare use', with prioritisation of the latter as it was considered a wider term that could include an estimate of the former. Although they did not make the final COS based on voting results, they were noted as important in some but not all studies and could be included as an additional outcome where appropriate.

*** Under the treatment outcomes, 'Carer burden' and 'Treatment burden' were individually excluded, but it was considered whether there should be a more generic outcome of 'Burden', with the narrative that the type of burden would be determined by the nature of the study. However, this was excluded by voting via email (62.5% votes).

Appendix 6: Metrics/ measurement tools identified from the literature for the outcomes included in the two COS.

**Outcomes**	**Metrics/ measurement tools used**
**Prevention of multimorbidity**	
Adverse events	Fatal (death, stroke), and non-fatal adverse events (hospitalisations, out-patient visits, surgeries etc.)
Comorbidity (development of new comorbidity)	Incidence of comorbidities
Health risk behaviour	Self-report measures of behaviour e.g., adherence to diet, exercise, medication etc.<br>Biochemical measures: Lipid profile, blood pressure, HbA1c etc.<br>Anthropometrics: height, weight, waist circumference etc.
Quality of life (including Health-related quality of life)	Short Form Health Survey (SF-36)<br>Short Form Health Survey (SF-12)<br>European Quality of Life (EQ-5D)<br>Health Utilities Index (HUI-3)<br>Saint George Respiratory Questionnaire hospital (SGRQ)<br>European Organization for Research and Treatment of Cancer Quality of Life Questionnaire (QLQ-C30)
**Treatment of multimorbidity**	
Adherence to treatment	Self-report measures such as Morisky Medication Adherence Scale (MMAS-4), CASE Adherence Index, Treatment Adherence Questionnaire of Patients with Hypertension (TAQPH), Visual Analogue Scale (VAS)<br>Other measures: Sputum collection with negative/positive sputum results, Wisepill (real-time electronic adherence monitoring)
Adverse events	Mortality rate<br>Treatment Emergent signs and symptoms (TESS) score<br>Incidence of adverse events (e.g., self-reported (e.g., vomiting), and/or biochemically ascertained (e.g., vital signs, electrocardiogram, haematological measures etc.)
Healthcare cost (out-of-pocket cost of treatment)	Direct non-medical (participant time spent travelling to and attending appointments), and indirect costs (lost productivity associated with illness or premature mortality).
Quality of life (including Health-related quality of life)	Generic questionnaires: World Health Organization Quality of Life-short version, WHOQOL-BREF, Short Form Health Survey (SF-12), Medical Outcomes Study 36-item, Short Form Health Survey (SF-36), European Quality of Life five-dimensional questionnaire, Five-level version (EQ-5D-5L).<br>Disease specific questionnaires: Irritable Bowel Syndrome Quality of Life (IBS-QOL) questionnaire, Hypertension scale of the Quality-of-Life Instruments for Chronic Diseases (QLICD-HY).
